# Breaking the scaling relationship via thermally stable Pt/Cu single atom alloys for catalytic dehydrogenation

**DOI:** 10.1038/s41467-018-06967-8

**Published:** 2018-10-26

**Authors:** Guodong Sun, Zhi-Jian Zhao, Rentao Mu, Shenjun Zha, Lulu Li, Sai Chen, Ketao Zang, Jun Luo, Zhenglong Li, Stephen C. Purdy, A. Jeremy Kropf, Jeffrey T. Miller, Liang Zeng, Jinlong Gong

**Affiliations:** 1 Key Laboratory for Green Chemical Technology of Ministry of Education, School of Chemical Engineering & Technology, Tianjin University, Tianjin, 30072 P. R. China; 2 Collaborative Innovation Center for Chemical Science & Engineering (Tianjin), Tianjin, 30072 P. R. China; 3 Center for Electron Microscopy, Institute for New Energy Materials and Low-Carbon Technologies, School of Materials, Tianjin University of Technology, Tianjin, 300384 P. R. China; 4 Energy and Transportation Science Division, Oak Ridge National Laboratory, Oak Ridge, TN 37831 USA; 5 Davidson School of Chemical Engineering, Purdue University, West Lafayette, IN 47907 USA; 6 Chemical Technology Division, Argonne National Laboratory, Argonne, IL 60439 USA

## Abstract

Noble-metal alloys are widely used as heterogeneous catalysts. However, due to the existence of scaling properties of adsorption energies on transition metal surfaces, the enhancement of catalytic activity is frequently accompanied by side reactions leading to a reduction in selectivity for the target product. Herein, we describe an approach to breaking the scaling relationship for propane dehydrogenation, an industrially important reaction, by assembling single atom alloys (SAAs), to achieve simultaneous enhancement of propylene selectivity and propane conversion. We synthesize γ-alumina-supported platinum/copper SAA catalysts by incipient wetness co-impregnation method with a high copper to platinum ratio. Single platinum atoms dispersed on copper nanoparticles dramatically enhance the desorption of surface-bounded propylene and prohibit its further dehydrogenation, resulting in high propylene selectivity (~90%). Unlike previous reported SAA applications at low temperatures (<400 °C), Pt/Cu SAA shows excellent stability of more than 120 h of operation under atmospheric pressure at 520 °C.

## Abstract (web summary)

Enhancing the catalytic activity of noble-metal alloys is frequently accompanied by side reactions. Here, the authors describe an approach to break the scaling relationship for propane dehydrogenation, by assembling single atom alloys, to achieve simultaneous enhancement of propylene selectivity and propane conversion.

## Introduction

The development of new catalysts with simultaneously enhanced activity and selectivity is always the ultimate goal of researchers. In heterogeneous catalysis, the screening of new catalyst can be simply guided by the Sabatier principle. It indicates that the best catalysts should bind reactive intermediates with suitable interaction strength: neither too strong that leads to surface poisoning, nor too weak which fails to activate the reactants^1^. The principle behind this phenomenon can be attributed to the existence of scaling properties of adsorption energies on transition metal surfaces^2^, and various linear scaling relationships have been developed which connect the reaction energy/barriers with binding energy of representative surface species, i.e., descriptors. Therefore, the complicated reaction kinetics can be described by only a few descriptors, which enables fast screening of potential catalytic materials with the help of density functional theory (DFT) calculations^3^.

However, due to the existence of the scaling relationship, the enhancement of the activity toward the aimed product frequently accompanies a series of similar side reactions, leading to the reduced selectivity^4^. Considering the specific case of propane dehydrogenation (PDH), the key industrial reaction for the production of propylene is normally catalyzed by PtM alloys such as PtSn^5^, PtZn^6^, PtGa^7^, PtIn^8^, PtGe^9^, and PtCu^10^. Our previous DFT calculations indicate that the catalytic properties of these PtM alloys are indeed restricted by the scaling relationship, which normally results in the promoted selectivity to propylene accompanied with the suppressed intrinsic activity for dehydrogenation of propane^11^. Therefore, it is desirable to develop a catalyst that can break the scaling relationship to achieve the improvement of activity and selectivity concurrently^12^.

Single-atom catalysis has become one of the most active new frontiers in heterogeneous catalysis^13^. A large number of single-atom catalysts (SACs) exhibit distinct and often outstanding performances in a broad scope of chemical reactions, such as CO oxidation^14,15^, water-gas shift reaction (WGS)^16–18^, methanol steam reforming (MSR)^19,20^, hydrogen evolution reaction (HER)^21–23^, hydrogenation of substituted nitroarenes^24,25^, hydrogenation of alkynes and dienes^26–29^, and hydrogenation of carbon dioxide^30–32^. However, SACs do not find broad application in high temperature (> 500 °C) light alkane dehydrogenation reactions. Although single-site Zn(II)^33^, Co(III)^34^, Ni(II)^35^, and Ga(III)^36^ on silica have been developed as selective PDH catalysts, these active sites suffer from low activity compared to metallic sites. To the best of our knowledge, high stability and selectivity have not been achieved for the PDH reaction with the use of single-atom Pt catalysts.

Herein, we report that Pt/Cu single atom alloy (SAA) breaks the PtM alloy scaling relationship during the PDH, with a  great improvement of propylene selectivity and a  slight loss of intrinsic dehydrogenation activity. Based on the theoretical analysis, we design and synthesize the catalysts with Pt/Cu SAA supported on γ-alumina via an atomic dilution method and confirm the formation of Pt/Cu SAA through detailed characterizations, such as CO-diffuse reflectance infrared Fourier-transform spectroscopy (CO-DRIFTS), aberration-corrected high-angle annular dark-field scanning transmission electron microscope (AC-HAADF-STEM) and extended X-ray absorption fine structure (EXAFS) spectra. Subsequent catalytic tests demonstrate the transferability from DFT calculations to practical catalytic reaction and the Pt/Cu SAA catalyst with the low loading of Pt (0.1 wt%) displays propylene selectivity of ~90% with the high formation rate of 10.6 mol g^−1^_Pt_ h^−1^ under the conditions of atmospheric pressure, 520 °C, WHSV = 4 h^−1^, C_3_H_8_/N_2_ = 1/1. Unlike previous reported low temperature SAA applications^26,29,37–39^, our synthesized Pt/Cu SAA shows excellent stability for at least 120 h on stream at 520 °C.

## Results

### Theoretical prediction

Previous studies have shown that the alkane dehydrogenation reaction is insensitive to the structure of the Pt particles, because single atoms of Pt are capable of catalyzing the reaction^40^. However, undesired side reactions that occur during the alkane dehydrogenation, such as hydrogenolysis, isomerization and coke formation, are structure sensitive reactions, requiring more than one Pt atom^41^. Therefore, isolated Pt atoms are expected to be a potential active center with high propylene selectivity. It is worth noting that noble metals are active for dehydrogenation in the metallic state^41^. Xiong et al. reported that single Pt atoms on CeO_2_ can only promote the cleavage of C–C bonds of hydrocarbons with no observed selectivity toward dehydrogenation^42^. Therefore, SAAs attract our attention, where the catalytically active metal is atomically dispersed in the surface layer of the more inert host metal and exists in the metallic state, distinguishing from single atoms positively charged on metal oxides^26,43^. Such SAAs have been widely used as a strategy for selective hydrogenation reactions, such as the selective hydrogenation of styrene, acetylene, and 1,3-butadiene^26,29^. Recently, Pt/Cu SAAs were suggested as an approach to coke-resistant C–H activation chemistry and examined in the systems of methyl groups, methane, butane, and propane^37^. However, no stable SAA under reducing environment at high temperatures (>400 °C) has been reported so far. This raises the question whether SAAs are capable of breaking the restriction of the scaling relationship for PtM alloys and catalyzing dehydrogenation reactions at high temperatures stably.

**Fig. 1:**
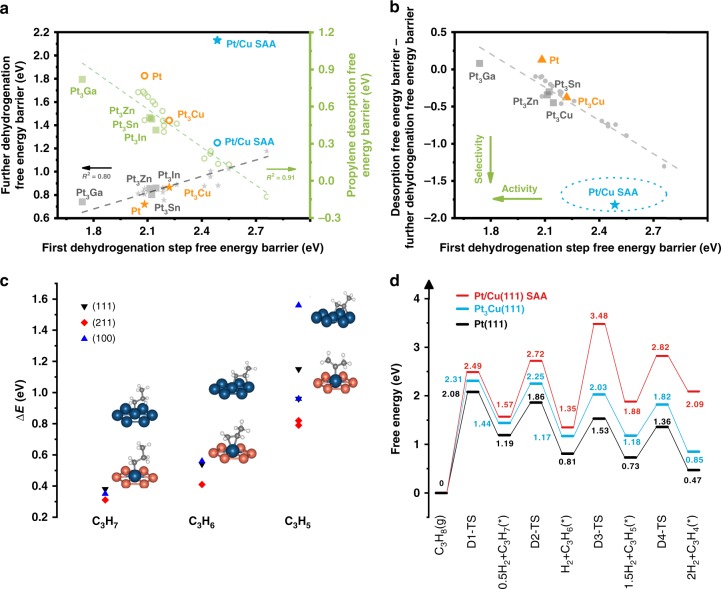
Pt/Cu SAA breaking the PtM alloy scaling relationship for PDH. **a** Scaling relationship between first dehydrogenation barrier and propylene desorption barrier/deep dehydrogenation barrier (highest barrier from di-σ propylene to C_3_H_4_), data for the Pt_3_M alloy systems are obtained from ref. ^11^. **b** Screening of Pt-based bimetallic catalyst for PDH, data for the Pt_3_M alloy systems are obtained from ref. ^11^. **c** Binding energy difference and structures (on (111) surface only) of dehydrogenated intermediates C_3_H_*x*_ (*x* = 7, 6, 5) over pure Pt surfaces and Pt/Cu SAA surfaces. The ΔE is defined as *E*_ads_(on Pt/Cu SAA surfaces)−*E*_ads_(on Pt surfaces). Color: Pt—blue; Cu—orange; C—gray; H—white. **d** Energy profiles of PDH over Pt/Cu SAA, Pt_3_Cu(111) and Pt(111)

As shown in previous theoretical studies, the PDH activity can be described by the first dehydrogenation barrier which is the rate-determining step, and the selectivity was estimated by the difference between di-σ propene desorption barrier and its further dehydrogenation barrier (the highest barrier for di-σ propene dehydrogenation to C_3_H_4_ shown in Fig. 1d and Supplementary Figure 1)^11^. Due to the existence of the scaling relationship for Pt_3_M (M = 3d and 4d transition metals), low dehydrogenation barrier (i.e., high PDH activity) accompanies with strong C–Pt interaction, leading to strong propene adsorption (i.e., low propene selectivity) (Fig. 1a). However, isolated Pt atoms dispersed in Cu nanoparticles maintain a reasonable dehydrogenation activation barrier and at the same time display a quite high selectivity, which is reflected by the low binding strength of propylene and high deep dehydrogenation barrier (Fig. 1a). Our DFT calculations suggest that Pt/Cu SAA indeed breaks the PtM alloy scaling relationship, which is different from the scaling for conventional Pt alloys during the PDH (Fig. 1b). Unlike over Pt surfaces, the isolated Pt atom in Pt/Cu SAA can only interact with a single Pt atom for the deep dehydrogenated C_3_H_5_, a model precursor of coke formation, instead of more stable three Pt–C interactions on threefold hollow site over Pt(111) (Fig. 1c). The deep dehydrogenation thus becomes strongly endothermic over Pt/Cu SAA with a significant free energy barrier (>2 eV), compared with the same step over Pt(111) with a surmountable barrier (<1 eV), leading to a much higher propylene selectivity over Pt/Cu SAA (Fig. 1d). The binding strength of π adsorbed propylene over Pt/Cu SAA (−0.57 eV) is also weaker than the di-σ ones over Pt_3_Cu(111) (−0.88 eV) and Pt(111) (−1.10 eV), further facilitating propylene desorption over Pt/Cu SAA (Supplementary Table 1). However, for the first two dehydrogenation steps, the geometry effects mentioned above are less apparent, due to no binding mode change for propyl over our calculated surfaces. Therefore, the destabilization of dehydrogenated intermediates over Pt/Cu SAA is less obvious than C_3_H_5_ (Fig. 1c). This is also important for Pt/Cu SAA to maintain reasonably low dehydrogenation barriers for the first two dehydrogenation steps. Compared with Pt(111), Pt/Cu SAA only slightly increases the corresponding dehydrogenation barriers by ~0.4 eV, resulting in similar PDH activity over pure Pt and Pt/Cu SAA (Fig. 1d). Note that our convergence test suggests Pt/Cu(111) surface model can well describe Pt/Cu SAA nanoparticles which are larger than 2.1 nm in diameter (Pt/Cu_404_, Supplementary Figure 2). Similar behavior has also been observed for single Pt atom over Cu(100) and Cu(211) surfaces (Supplementary Figure 3).

DFT results also identify that the exposed isolated surface Pt atoms within Cu(111) facets of Pt/Cu SAA nanoparticles (larger than 2.1 nm in diameter) are thermodynamically more stable than other types of Pt species: (1) isolated subsurface Pt atoms, (2) single Pt atoms within Cu(100) facets, (3) single Pt atoms at low coordinated step edge and corner sites, and (4) surface dimers within Cu(111) (Supplementary Tables 2–4). Therefore, isolated Pt atoms within Cu(111) facets are thermodynamically preferred in the case with high Cu to Pt molar ratio, which maximizes the utilization of Pt atoms.

Therefore, it is reasonable to speculate that the catalysts based on Pt/Cu SAA have the potential to achieve a decent catalytic performance for PDH. Note that we also calculated the relative stability of Pt/Ag SAA surfaces, and the single Pt atom is always slightly more stable at subsurface instead of surface. Although the dehydrogenation barrier is similar for Pt/Cu(111) and Pt/Ag(111) SAA surfaces (Supplementary Figure 4), the catalytic activity for Pt/Ag(111) might be low due to the less exposed surface Pt atoms over Ag.

### Preparation of Pt/Cu SAA via atomic dilution

We simply synthesized the catalysts with Pt/Cu SAA supported on γ-alumina by means of atomic dilution. Only diffraction lines due to γ-alumina were detected in the XRD patterns of the catalysts after calcination (Supplementary Figure 5a), suggesting Cu oxide species are highly dispersed on the surface of alumina. When treated under H_2_-rich condition at 600 °C for 1 h, the oxide species of Pt and Cu on γ-alumina can be fully reduced since the highest reduction peak temperature for PtCu bimetallic catalysts is below 200 °C (Supplementary Figure 6). For the catalysts after reduction, the diffraction line of Cu(111) becomes apparent as the content of Cu increases to 6.7 wt%, demonstrating the formation of Cu nanoparticles on alumina (Supplementary Figure 5b). Given the appearance of the co-reduction peaks (Supplementary Figure 6), the close contact between Cu oxide species and Pt oxide species  leads to preferential formation of PtCu bimetallic nanoparticles rather than two metal phases apart from each other during reduction. The PtCu bimetallic nanoparticles formed on γ-Al_2_O_3_ during reduction in the H_2_-rich atmosphere at high temperatures can be considered as a Pt/Cu surface alloy, as Pt is preferentially dispersed on the surface layer of PtCu alloys^29,44^. Then with the amount of Pt fixed, further addition of Cu lowers the concentration of Pt in the Pt/Cu surface alloy leading to easier formation of Pt/Cu SAA. It can be expected that Pt atoms would be individually dispersed in Cu nanoparticles if diluted to a certain degree and the structure would stay the same with further dilution.

**Fig. 2:**
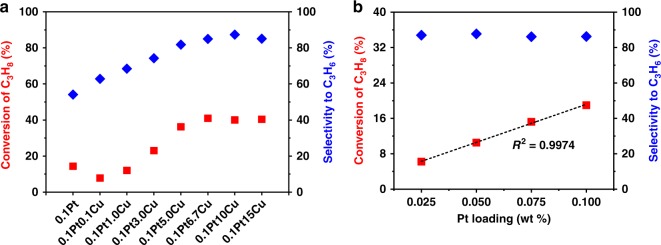
Initial activity as a function of Pt/Cu atom ratio. **a** The initial activities of the catalysts with fixed amount of Pt (0.1 wt%) and increasing content of Cu (0, 0.1, 0.3, 3, 5, 6.7, 10, and 15 wt%). With the Cu/Pt atom ratio increasing, the initial conversion of propane first rises and then remains unchanged, implying a critical Cu/Pt molar ratio for the formation of Pt/Cu SAA. **b** The initial activities of the catalysts with fixed amount of Cu (10 wt%) and progressively decreasing content of Pt (0.1, 0.075, 0.05, and 0.025 wt%). The excellent linear correlation between the initial activity and Pt concentration supports the formation of Pt/Cu SAA. Catalytic conditions: atmospheric pressure, 600 °C, C_3_H_8_/H_2_ = 1/1, with balance N_2_ for total flow rate of 50 mL min^−1^, WHSV of propane = 4 h^−1^ and 250 mg of sample (**a**) or WHSV of propane = 40 h^−1^ and 25 mg of sample (**b**)

To verify the above conjecture, we compared the initial activities of the catalysts with fixed amount of Pt (0.1 wt%) but various contents of Cu (Fig. 2a). As expected, the initial conversion of propane increases  until the content of Cu reaches 6.7 wt% and then remains unchanged with the amount of Cu further increasing to 15 wt%. Hence, we presume that the formation of Pt/Cu SAA on γ-alumina can be achieved for the catalysts with the addition of Cu no less than 6.7 wt%. Furthermore, given the assumption that Pt atoms are individually dispersed in Cu nanoparticles for 0.1Pt10Cu/Al_2_O_3_, keeping the content of Cu (10 wt%) unchanged, we decreased progressively the amount of Pt from 0.1 wt% to 0.025 wt%, which guarantees the structure unchanged. The initial activity correlates linearly with the Pt content, which again suggests the formation of Pt/Cu SAA (Fig. 2b). Note that the intersection of the fitting line with *Y*-axis is not zero, which is attributed to the weak catalytic ability of γ-alumina-supported Cu.

### Formation of Pt/Cu SAA

**Fig. 3:**
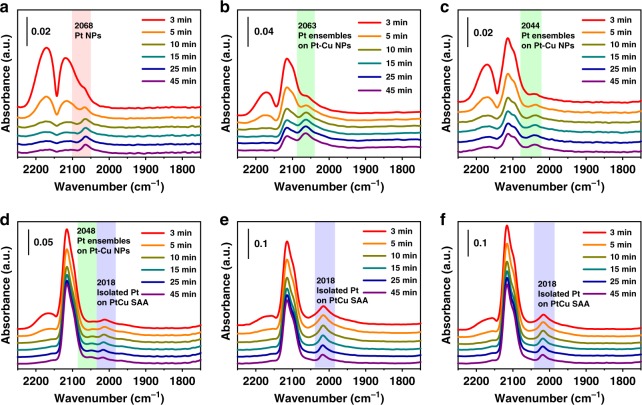
In situ CO-DRIFTS for the catalysts with fixed Pt but increasing Cu contents. **a** 0.1Pt/Al_2_O_3_, **b** 0.1Pt0.1Cu/Al_2_O_3_, **c** 0.1Pt0.3Cu/Al_2_O_3_, **d** 0.1Pt3Cu/Al_2_O_3_, **e** 0.1Pt6.7Cu/Al_2_O_3_ and **f** 0.1Pt10Cu/Al_2_O_3_. As Pt is continuously diluted with Cu, the band of CO linearly adsorbed on Pt firstly redshifts from 2068 cm^−1^ to 2044 cm^−1^ and then an exclusively band at 2018 cm^−1^ appears and remains unchanged with further increasing the Cu content. The band at 2018 cm^−1^ is ascribed to CO linearly adsorbed on single atoms of Pt in the metallic state

The structure of Pt/Cu SAA supported on γ-alumina was confirmed by CO-DRIFTS, AC-HAADF-STEM images and EXAFS spectra. As shown in the in situ DRIFTS, the weak bands caused by bridge-adsorbed CO on two adjacent Pt atoms appear at around 1835 cm^−1^ for 0.1Pt/Al_2_O_3_ and 0.1Pt0.1Cu/Al_2_O_3_ (Supplementary Figure 7a, b), indicating the existence of dimers or clusters of Pt^45^. Meanwhile, the lack of the band of bridge-bonded CO on other catalysts suggests the exclusively isolated Pt atoms or low concentrations of bridging CO^46,47^. For 0.1Pt/Al_2_O_3_, Fig. 3a shows a main band at 2068 cm^−1^, which can be assigned as linearly adsorbed CO on Pt nanoparticles. For CO adsorption on 0.1Pt0.1Cu/Al_2_O_3_, 0.1Pt0.3Cu/Al_2_O_3_, and 0.1Pt3Cu/Al_2_O_3_, the bands due to linearly bonded CO on Pt ensembles on PtCu bimetallic nanoparticles appear at 2063–2044 cm^−1^ (Fig. 3b–d). The unique band centered at 2018 cm^−1^ emerges in the spectra of 0.1Pt3Cu/Al_2_O_3_, 0.1Pt6.7Cu/Al_2_O_3_, and 0.1Pt10Cu/Al_2_O_3_ (Fig. 3d–f), which can be attributed to linearly bonded CO on single atoms of Pt in the metallic state^48^, differing from those at 2080–2115 cm^−1^ ascribed to CO linearly adsorbed on Pt^δ+^ single-atom sites on metal oxides^45,49–51^. Note that our DFT calculations predict the top-adsorbed CO stretching frequencies are 2061 cm^−1^ over Pt(111) and 2021 cm^−1^ over Pt/Cu SAA, which are very close to experimentally observed locations (Supplementary Table 5). Combining the fact that there are no bands due to CO on Pt ensembles detected for 0.1Pt6.7Cu/Al_2_O_3_ and 0.1Pt10Cu/Al_2_O_3_, it can be inferred that Pt atoms on the surface of Cu nanoparticles are individually dispersed. It is worth pointing out that the variation trend of CO adsorption behavior is highly consistent with that of the initial activities of the catalysts as the Cu content increases.

**Fig. 4:**
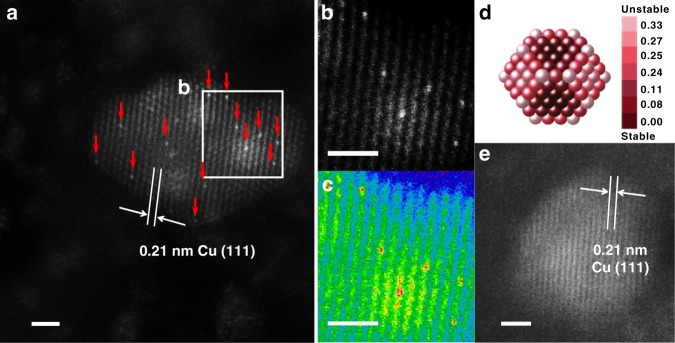
Morphology of γ-Al_2_O_3_-supported Pt/Cu SAA. **a** HAADF-STEM images with typical region of the reduced 0.1Pt10Cu/Al_2_O_3_ catalyst, showing Pt atoms individually dispersed on Cu(111). Pt atoms are highlighted by red arrows. **b**, **c** The enlarged image and the colored intensity map from the selected region in **a**. The lattice spacing of Pt/Cu SAA is 0.21 nm, which is consistent with that of Cu(111). **d** Relative stability of Pt single atoms over Cu (~2.1 nm) nanoparticle: dark red indicates stable location of Pt atom. Color bar unit is eV/Pt atom. **e** HAADF-STEM images with typical region of the reduced 10Cu/Al_2_O_3_ catalyst, showing no Pt atoms existing on the surface of Cu nanoparticles. Scale bars, 1 nm (**a**), (**b**), (**c**) and (**e**)

The structure of Pt/Cu SAA was further addressed by AC-HAADF-STEM images. Single Pt atoms can be distinguished from Cu atoms due to differences in the Z-contrast^29,52^. Figure 4a indicates the presence of individual brighter Pt atoms in Cu nanoparticles. As a contrast, we failed to find Pt atoms over the 10Cu/Al_2_O_3_ catalyst (Fig. 4e and Supplementary Figure 11). The lattice spacing of Pt/Cu SAA is 0.21 nm, which is in good consistence with the lattice spacing of Cu(111), indicating dilute dispersion of Pt atoms in Cu nanoparticles. Single Pt atoms in Cu nanoparticles are repeatedly observed in different regions of the reduced 0.1Pt10Cu/Al_2_O_3_ catalyst, and no clusters or nanoparticles of Pt on γ-alumina are found (Supplementary Figures 9, 10, and 2,3). In terms of 0.1Pt/Al_2_O_3_, the Pt nanoparticles are dispersed on γ-alumina with an average particle size of about 2.3 nm (Supplementary Figure 8). In situ EXAFS performed at the Pt–L_III_ edge and Cu–K edge reveals the coordination of Pt and Cu in monometallic Pt and bimetallic PtCu catalysts (Supplementary Figures 12–19 and Table 7). At the Pt-L_III_ edge, there are ∼6.8 Pt–Cu bonds at 2.53 Å and no Pt–Pt coordination is detected in the fully reduced 0.1Pt6.7Cu/Al_2_O_3_ catalyst, providing direct evidence for the isolated Pt atoms in the bimetallic PtCu catalysts at high Cu to Pt molar ratios. Due to the low Pt loading, at the Cu–K edge, there are only 7.0 Cu–Cu bonds at 2.55 Å consistent with 2 nm metallic Cu nanoparticles. For the 0.1Pt/Al_2_O_3_ catalyst, the existence of Pt–Pt coordination indicates the formation of Pt islands and/or clusters.

### C–H activation studies

**Fig. 5:**
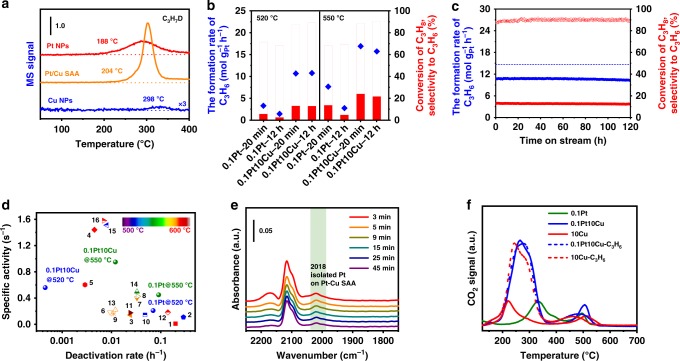
Catalytic performances and characterizations of spent catalysts. **a** Signals of C_3_H_7_D during TPSR over 0.3Pt/Al_2_O_3_, 0.1Pt10Cu/Al_2_O_3_ and 10Cu/Al_2_O_3_ for P–D scrambling. The mass ratio of 45/44 represents the level of C_3_H_7_D. Compared with Pt nanoparticles, a little higher temperature of C–H activation for Pt/Cu SAA suggests a slightly weaker intrinsic activity of dehydrogenation. **b** Catalytic performances at the initial and final period at 520 °C and 550 °C during 12 h PDH, showing significantly improved performances of Pt/Cu SAA compared with Pt nanoparticles. **c** Long-term stability test for 0.1Pt10Cu/Al_2_O_3_ at 520 °C for 120 h, demonstrating its excellent stability. The thermodynamic conversion calculated as mole g^−1^_Pt_ h^−1^ is presented (dash line). **d** Normalized activities of the formation rate of C_3_H_6_ by specific content of Pt for the catalysts described in this work and in the literature (1–16, see Supplementary Table 10). **e** CO-DRIFTS of 0.1Pt10Cu/Al_2_O_3_ after long-term stability test, showing the maintainance of the sturcture of Pt/Cu SAA. **f** Mass-spectra signals of CO_2_ during TPO experiments for the spent catalysts of 0.1Pt/Al_2_O_3_, 0.1Pt10Cu/Al_2_O_3_, and 10Cu/Al_2_O_3_ after 12 h PDH at 520 °C and for the catalysts of 0.1Pt10Cu/Al_2_O_3_ and 10Cu/Al_2_O_3_ with treatment in the flow of C_3_H_6_/N_2_/H_2_ = 1/41/8 mL min^−1^ at 520 °C. The TPO profile of Pt/Cu SAA is very close to that of Cu nanoparticles, verifying its excellent anti-coke capability. Catalytic test conditions: atmospheric pressure, WHSV propane = 4 h^−1^, 250 mg of sample, C_3_H_8_/H_2_ = 1/1, with balance N_2_ for total flow rate of 50 mL min^−1^

Temperature-programmed surface reaction (TPSR) analysis was used to evaluate the intrinsic activity of the catalysts for PDH. We examined the TPSR over γ-alumina-supported Pt nanoparticles, Pt/Cu SAA, and Cu nanoparticles for propane–deuterium isotope scrambling (P–D scrambling) to compare their intrinsic ability to activate the C–H bonds of propane^53,54^. The C–H activation starts at about 188 °C on Pt nanoparticles, 204 °C on Pt/Cu SAA, and 298 °C on Cu nanoparticles, respectively (Fig. 5a). Moreover, according to the Pt dispersion (Supplementary Table 8) and the specific activity at 520 °C (Supplementary Table 9), the calculated TOF is 0.72 s^−1^ for 0.1Pt/Al_2_O_3_ and 0.56 s^−1^ for 0.1Pt10Cu/Al_2_O_3_. Note that the TOF value is quite close to that reported for Pt nanoparticles supported on calcined hydrotalcite^53^. Combining the results of TPSR of P–D scrambling and TOFs, we conclude that the intrinsic activity of Pt/Cu SAA for PDH is only slightly lower than that of Pt nanoparticle while Cu nanoparticles presents an extremely low capacity for C–H activation. This supports the slightly increased dehydrogenation barrier for Pt/Cu SAA compared with Pt(111) identified in the DFT studies.

### Catalytic performance

We examined the catalytic performances of 0.1Pt/Al_2_O_3_ and 0.1Pt10Cu/Al_2_O_3_ for PDH to demonstrate the transferability from DFT calculations to practical catalytic reaction. During the PDH at 520 °C, the propylene formation rate dramatically dropped from 3.7 to 1.6 mol g^−1^_Pt_ h^−1^ for 0.1Pt/Al_2_O_3_ after 12 h on stream (Fig. 5b). As a contrast, we did not observe deactivation for 0.1Pt10Cu/Al_2_O_3_ upon 12 h on stream (Fig. 5b) and the propylene formation rate only slightly decreased from 10.6 to 10.3 mol g^−1^_Pt_ h^−1^ even after 120 h on stream (Fig. 5c). The initial propylene formation rate for 0.1Pt10Cu/Al_2_O_3_ is 3.2 times of that for 0.1Pt/Al_2_O_3_, at the same time the selectivity to propylene improves to 90% for 0.1Pt10Cu/Al_2_O_3_ at 13.1% conversion compared with 70% for 0.1Pt/Al_2_O_3_ at 5.8% conversion. Although DFT calculations predict slightly increased dehydrogenation barrier for Pt/Cu SAA, its higher propylene formation rate can be attributed to its better Pt dispersion compared with 0.1Pt/Al_2_O_3_ (Pt dispersion = 29%). In terms of deactivation, a first-order deactivation model is used to estimate the catalyst stability^55^. The low deactivation rate of 0.0005 h^−1^ for 0.1Pt10Cu/Al_2_O_3_ quantitatively demonstrates its high stability when compared to 0.07 h^−1^ for 0.1Pt/Al_2_O_3_ (Supplementary Table 9). To further test the stability of the catalysts, the reaction temperature was increased to 550 °C (Fig. 5b and Supplementary Figure 24). During 12 h PDH, the formation rate of propylene dropped from 7.7 to 2.8 mol g^−1^_Pt_ h^−1^ for 0.1Pt/Al_2_O_3_, corresponding to the deactivation rate of 0.091 h^−1^. While for 0.1Pt10Cu/Al_2_O_3_, the formation rate of propylene only decreased from 16.8 to 15.8 mol g^−1^_Pt_ h^−1^ and the deactivation rate became 0.012 h^−1^, verifying its higher activity and better coke resistance. In addition, we examined the performance of 0.1Pt10Cu/Al_2_O_3_ for successive oxidation–reduction cycles. The conversion and selectivity of the catalyst had no change during five cycles at 520 °C (Supplementary Figure 25), demonstrating its good stability of dehydrogenation and regeneration. The initial activity of the catalyst can be largely restored after regeneration at 600 °C (Supplementary Figure 28), further confirming its cycle stability. The activity of the 10Cu/Al_2_O_3_ catalyst for PDH was very low, which can be neglected compared to that of the 0.1Pt10Cu/Al_2_O_3_ catalyst (Supplementary Figure 31).

We prepared the Pt/Ag SAA catalyst according to similar atomic dilution method mentioned above. The TPSR of P–D scrambling experiment indicates similar C–H activation ability between Pt/Ag SAA and Pt/Cu  SAA (Supplementary Figure 20a). However, under reaction conditions, the propane conversion for Pt/Ag SAA is only 32% at 600 °C, which is lower than 41% for Pt/Cu SAA, consistent with the DFT predicted trends (Supplementary Figure 20b and Supplementary Table 6).

### Spent catalysts

The spent catalysts were characterized to further shed light on the relationship between the improved reaction rate and stability and the structure of Pt/Cu SAA. DRIFTS of CO on the spent 0.1Pt10Cu/Al_2_O_3_ (Fig. 5e) show that surface Pt atoms are still individually dispersed even after a long-term stability test for 120 h on stream at 520 °C, which suggests the atomic dispersion of Pt in Cu is maintained during the PDH reaction. The slightly increased particle size of the bimetallic nanoparticles on Al_2_O_3_ after reaction indicates the thermal stability of the catalyst at 520 °C (Supplementary Figure 32). When the reaction temperature was raised to 600 °C, the sintering of copper nanoparticles was obvious (Supplementary Figure 3,3), which may contribute to the quick deactivation of 0.1Pt10Cu/Al_2_O_3_ during the dehydrogenation reaction at 600 °C. In addition, HAADF-STEM images of 0.1Pt10Cu/Al_2_O_3_ after successive oxidation–reduction cycles show that Pt atoms identified from their higher brightness comparing to their surrounding area are still individually dispersed in Cu nanoparticles, confirming its good cycle stability (Supplementary Figures 26, 27, 29, and 30). Regrettably, due to the lack of the EXAFS information for the regenerated 0.1Pt10Cu/Al_2_O_3_, the sintering of partial platinum cannot be fully ruled out. Considering that coke deposition is another reason for catalyst deactivation during propane dehydrogenation^41^, the information on the coke formed on the surface of 0.1Pt/Al_2_O_3_, 0.1Pt10Cu/Al_2_O_3_, and 10Cu/Al_2_O_3_ were collected. The Raman spectra (Supplementary Figure 21) show that the *I*_D_/*I*_G_ ratio increases in the order of 0.1Pt/Al_2_O_3_ (0.76) < 0.1Pt10Cu/Al_2_O_3_ (0.82) < 10Cu/Al_2_O_3_ (0.86), suggesting a reverse order of dehydrogenation degree of the coke^56^. The temperature-programmed oxidation (TPO) profiles (Fig. 5f) indicate that there are two types of coke on each spent catalyst because of two peaks in each profile. We attribute the small high-temperature peaks centered at around 500 °C to the combustion of the hard coke deposited on the support. While the main low-temperature peaks are described as the combustion of the soft coke formed on the metal, of which the temperatures decreased in the order of 0.1Pt (335 °C) > 0.1Pt10Cu (265 °C) > 10Cu (225 °C)^56,57^. The good consistence between the dehydrogenation degree of the coke and the burning temperature of the soft coke on the metal implies that the deep dehydrogenation reactivity decreases in the order of Pt nanoparticles, Pt/Cu SAA, and Cu nanoparticles, which corroborates the trend of deep dehydrogenation barrier identified by the DFT calculations. Moreover, it is worth noting that the target propylene as coke precursor can partly transform into carbon deposits with the help of Cu, which accounts for the formation of coke on Pt/Cu SAA. As shown in Fig. 5f, after treating in the flow of propylene for the same time, the TPO profile of 0.1Pt10Cu/Al_2_O_3_ is similar to that of 10Cu/Al_2_O_3_, confirming the coke resistance of 0.1Pt10Cu/Al_2_O_3_ comparable with that of 10Cu/Al_2_O_3_. The temperature-programmed desorption (TPD) of propylene (Supplementary Figure 22) indicates that the interaction between the propylene and metal sites is weaker for Cu nanoparticles and Pt/Cu SAA than that for Pt nanoparticles. This weak interaction minimizes carbon deposits on the active Pt sites to help remain the activity during reaction.

## Discussion

Our DFT calculation shows that different from the conventional Pt alloys, the Pt/Cu SAA breaks the PtM alloy scaling relationship during PDH, displaying a quite negative difference between the desorption energy and further dehydrogenation barrier of propylene, and at the same time maintaining a reasonable dehydrogenation energy barrier for propane. We further synthesized a kind of SAA catalysts with single Pt atoms dispersed on Cu and Ag nanoparticles through an atom dilution method. In the catalytic dehydrogenation of propane, the Pt/Cu SAA catalyst with the low loading of Pt (0.1 wt%) displays propylene selectivity of ~90% with the high formation rate of 10.6 mol g^−1^_Pt_ h^−1^ under the conditions of atmospheric pressure, 520 °C, WHSV = 4 h^−1^, C_3_H_8_/N_2_ = 1/1 and shows excellent stability for at least 120 h on stream. Our study highlights the transferability from the DFT calculations to the realistic catalytic system and implies isolated Pt atoms in the metallic state as promising active sites for alkane dehydrogenation at high temperatures.

## Methods

### Computational details

All the total energy self-consistent calculations were carried out in Vienna ab initio simulation package (VASP)^58^ using the generalized gradient approximation with the Bayesian error estimation functional with van der Waals corrections (BEEF-vdW)^59,60^. We used the projector augmented-wave (PAW) method to handle the ionic-core interaction^61^. The valence wave functions were expanded by plane-wave with a cutoff energy of 400 eV and the electronic tolerance value is 1 × 10^−4^ eV. A Methfessel–Paxton smearing with 0.15 eV width was employed to speed up the convergence and the total energies were evaluated by extrapolating to zero broadening^62^. The thickness of the employed (111) slabs, separated by >15 Å vacuum layer, are five layers, with top two layers relaxed on each surface. Optimized geometries were found when the force on each relaxed atom was <0.02 eV/Å. The Monkhorst–Pack^63^ k-points mesh of 3 × 3 × 1 was used in a 4 × 4 unit cell of each model slab. Test calculations indicate the numerical accuracy for the binding energies with finer k-points meshes is within 0.03 eV. We applied same parameters for the nanoparticle calculations, except the k-points grid was reduced to Γ point.

### Catalyst preparation

All the catalysts were prepared by incipient wetness co-impregnation method. H_2_PtCl_6_·6H_2_O (Chemart (Tianjin) Chemical Technology Co., Ltd, 99.9%) and Cu(NO_3_)_2_·3H_2_O (Alfa Aesar (China) Chemical Co., Ltd, 99.0%) were mixed and used as precursors and γ-Al_2_O_3_ (Sinopharm Chemical Reagent Co., Ltd, 98.0%) was used as support. After impregnation, the catalysts were placed in the atmosphere statically overnight and then dried in the flowing air at 80 °C for 12 h and then calcined at 600 °C for 2 h. The metal loading is based on the weight ratio between metal and γ-Al_2_O_3_.

### Characterization

Transmission electron microscope (TEM) images were taken using a JEOL JEM 2100 F system at an accelerating voltage of 200 kV equipped with a field emission gun. For the catalysts of 0.1Pt/Al_2_O_3_ and 0.1Pt10Cu/Al_2_O_3_, the sample was firstly reduced at 600 °C for 1 h in a stream of 18 vol% H_2_/N_2_. Then, the sample powder was dispersed in deionized water by ultrasonic and supported on a copper grid coated with an ultrathin holey carbon film.

The in situ diffuse reflectance infrared Fourier-transform spectroscopy (DRIFTS) experiments were performed on a Thermo Scientific Nicolet IS50 spectrometer, equipped with a Harrick Scientific DRIFTS cell fitted with ZnSe windows and a mercury–cadmium–telluride (MCT) detector cooled by liquid N_2_. The DRIFTS measurements were carried out for catalysts with fixed amount of Pt (0.1 wt%) and different content of Cu. The fresh and spent catalysts were heated from ambient temperature to 600 °C at a rate of 10 °C min^−1^ and retained at 600 °C in a flow rate of 50 mL min^−1^ of 20 vol% H_2_/Ar. Then, the catalysts were cooled down to 30 °C and the backgrounds (8 cm^−1^ resolution, 64 scans) were collected after Ar purging in a flow rate of 20 mL min^−1^ for at least 1 h. With the addition of a flow of 3 mL min^−1^ of CO, the adsorption of CO molecules on the surface of the catalysts continued for 30 min. After that, the DRIFTS spectra were recorded till no visible change in the absorption band intensities under Ar purging.

High-angle annular dark-field scanning transmission electron microscopy (HAADF-STEM) images were collected using a Titan Cubed Themis G2 300 (FEI) 200 kV aberration-corrected scanning transmission electron microscope (AC-STEM), capable of sub-angstrom resolution at Tianjin University of Technology. For the catalysts of 0.1Pt10Cu/Al_2_O_3_ and 10Cu/Al_2_O_3_, the sample was first reduced at 700 °C for 1 h in a stream of 18 vol% H_2_/N_2_. Then, the sample powder was dispersed in deionized water by ultrasonic and deposited on a molybdenum grid coated with an ultrathin holey carbon film.

Pt L3 edge (11.564 keV) XAS spectra were collected at the 10 ID beam line at the Advance Photo Source, Argonne National laboratory. Due to the low Pt loading, the Pt edge was measured in fluorescence mode. Since Cu has a fluorescence line near that of Pt and there is a large loading of Cu, an energy resolved fluorescence spectrometer using a bent crystal laue energy analyzer with soller slits and a Pilatus 100k silicon pixel detector was required to detect from the weak Pt fluorescence signal without saturating the detector with fluorescence form copper. Samples for XAS analysis were ground into a fine powder and pressed into a stainless-steel sample holder. Samples were treated in situ in a custom built reactor^64^ and heated to 550 °C in 3% H_2_ (balance He) for 30 min and then cooled to 100 °C for collection of spectra. Several spectra were averaged to give the final data used in analysis. There were no noticeable differences in the first and last spectra collected for each sample, demonstrating that the catalyst did not change over the course of measurement. Helium used was passed through a copper trap to remove trace oxygen impurities.

Cu–K edge spectra were collected at the 10BM beam line at the Advance Photo Source, Argonne national laboratory. Samples for XAS analysis were ground into a fine powder and pressed into a stainless-steel sample holder. Sample holders were placed in quartz tube reactors equipped with kapton windows and three way valves for gas flow. Transmission mode samples were treated in 3% H_2_ (balance He) at 550 °C for 30 min. The gas flow was then switched to pure He for 5 min to desorb surface hydrogen. The sample was then cooled in helium to room temperature and the reactor atmosphere was isolated using the three way valves.

Data analysis was performed using WinXAS 3.1 software. Phase and amplitude functions for Pt–Pt, Pt–O, and Cu–Cu scattering were extracted from experimental references. Pt–Pt and Cu–Cu scattering phase and amplitude was extracted from their respective foils (12 neighbors at 2.77 Å for platinum, 12 neighbors at 2.56 angstroms for Cu). Pt–O scattering phase and amplitude was extracted from Na_2_Pt(OH)_6_ (six neighbors at 2.05 angstroms). A phase and amplitude function for Pt–Cu scattering was created using FEFF using a single Pt–Cu pair with a bond distance of 2.66 angstroms. The amplitude reduction factor, absolute Debye–Waller factor and E0 correction for the Pt–Cu scattering pair was taken to be the same as the platinum foil, so that fit values of E0 and debye-waller factor for Pt–Cu scattering are relative to that of platinum foil.

Temperature-programmed experiments were all carried out with a Micromeritics AutoChem 2920 apparatus. For TPO measurements, 100 mg of sample was heated at 300 °C for 1 h and cooled down to 80 °C in flowing Ar (30 mL min^−1^) and then treated in flowing 10 vol% O_2_/He (20 mL min^−1^) at a rate of 10 °C min^−1^ up to 700 °C. The signal of CO_2_ was recorded by HIDEN QIC-20 mass spectrometer. For TPSR of P–D scrambling, 200 mg of sample was packed in a quartz tube. The sample was first treated by heating at a rate of 10 °C min^−1^ up to 600 °C and maintaining for 1 h in flowing D_2_ and then cooled down to 50 °C. After Ar purging for 30 min, the gas was switched to flowing 18 vol% C_3_H_8_/N_2_ and the system was purged for 30 min. Subsequently, the temperature of the sample was raised to 400 °C at a rate of 5 °C min^−1^ and the signals at *m*/*z* of 45, 44, and 4 were monitored by the mass spectrometer.

Turnover frequency (TOF) was calculated as moles of propylene formed per mole of exposed Pt per second:

1 $${\mathrm{TOF}} = R_{{\mathrm{C3H6}}}/D_{{\mathrm{Pt}}}$$

where *R*_C3H6_ is the specific activity of propylene formation (s^−1^) (Supplementary Table 8) and *D*_Pt_ is the dispersion of Pt.

### Catalytic testing

Catalytic tests were performed in a quartz fixed-bed reactor with 8 mm inner diameter and 24 cm length at atmosphere pressure. A volume of 250 mg of the calcined catalyst with particle size of 20–40 mesh was packed inside the quartz tubular reactor. The sample was first heated to 600 °C at a rate of 10 °C min^−1^ and retained at 600 °C for 1 h in flowing 18 vol% H_2_/N_2_. Afterward, a mixture of C_3_H_8_, H_2_, and N_2_ (8:8:34 vol%) was fed at a rate of 50 mL min^−1^. The weight hourly velocity (WHSV) of propane was around 4 h^−1^. The gas products were analyzed by an online GC (2060) equipped with a flame ionization detector (Chromosorb 102 column) and a thermal conductivity detector (Al_2_O_3_ Plot column). The propane conversion and selectivity to propylene were calculated from Eq. (2) and Eq. (3), respectively:

2 $${\mathrm{Con}}\left( \% \right) = 100 \times \left( {\left[ {{{F}}_{\mathrm{C3H8}}} \right]_{\mathrm{inlet}} - \left[ {{{F}}_{\mathrm{C3H8}}} \right]_{\mathrm{outlet}}} \right)/\left[ {{{F}}_{\mathrm{C3H8}}} \right]_{\mathrm{inlet}}.$$

3 $${\mathrm{Sel}}\left( \% \right) = 100 \times \left[ {{{F}}_{\mathrm{C3H6}}} \right]_{\mathrm{outlet}}/\left( {[{{F}}_{\mathrm{C3H8}}]_{\mathrm{inlet}} - [{{F}}_{\mathrm{C3H8}}]_{\mathrm{outlet}}} \right).$$

Where *F*_C3H8_ and *F*_C3H6_ means mole flow rate of propane and propylene. A first-order deactivation model was used to evaluate the catalyst stability:

4 $$k_{\mathrm{d}} = \left( {{\mathrm{ln}}{\mathrm{ }}\left[ {\left( {1{\mathrm{ }} - X_{\mathrm{final}}} \right){\mathrm{ }}/X_{\mathrm{final}}} \right]-{\mathrm{ln}}{\mathrm{ }}\left[ {\left( {1{\mathrm{ }}-X_{\mathrm{initial}}} \right)/X_{\mathrm{intial}}} \right]} \right)/t$$

where *X*_intial_ and *X*_final_, respectively, represent the conversion measured at the initial and final period of an experiment, and *t* represents the reaction time (h), *k*_d_ is the deactivation rate constant (h^−1^). High *k*_d_ value means rapid deactivation, that is, low stability. The mean catalyst life (*τ*) represents the time required for rates to decrease by e^−1^, and is estimated with the reciprocal of the deactivation rate constants.

## Electronic supplementary material

Supplementary Information

## Data Availability

The data that support the findings of this study are available from the corresponding author upon request.
