# Supplementary material for: Breaking the scaling relationship via thermally stable Pt/Cu single atom alloys for catalytic dehydrogenation
Source: Nat Commun. 2018 Oct 26;9:4454. doi: 10.1038/s41467-018-06967-8 (PMC6203812; doi:10.1038/s41467-018-06967-8)
Supplement: Supplementary file 1 — Supplementary Information [file 41467_2018_6967_MOESM1_ESM.pdf]

Supporting Information

Breaking the Scaling Relationship via Thermally Stable Pt/Cu Single Atom Alloys for Catalytic Dehydrogenation

Guodong Sun[#1,2], Zhi-Jian Zhao[#1,2], Rentao Mu[1,2], Shenjun Zha[1,2], Lulu Li[1,2], Sai Chen[1,2], Ketao Zang[3], Jun Luo[3], Zhenglong Li[4], Stephen C. Purdy,[5] Jeremy Kropf,[6] Jeffrey T. Miller,[5] Liang Zeng,[1,2] and Jinlong Gong[*1,2]

[1] Key Laboratory for Green Chemical Technology of Ministry of Education, School of Chemical Engineering & Technology, Tianjin University, Tianjin 30072, P. R. China.

[2] Collaborative Innovation Center for Chemical Science & Engineering (Tianjin), Tianjin 30072, P. R. China.

[3]Center for Electron Microscopy, Institute for New Energy Materials and Low-Carbon Technologies, School of Materials, Tianjin University of Technology, Tianjin 300384, P. R. China.

[4] Energy and Transportation Science Division, Oak Ridge National Laboratory, Oak Ridge, TN 37831, USA.

[5] Davidson School of Chemical Engineering, Purdue University, West Lafayette, IN 47907, USA.

[6] Chemical Technology Division, Argonne National Laboratory, Argonne, IL 60439, USA.

# These authors contributed equally to this work.

*** Corresponding Author**

E-mail: jlgong@tju.edu.cn.

Contents

1. **Methods for characterization……………………………………………………….…4**
2. **Results of extended X-ray absorption fine structure (EXAFS)…………………….….5**
3. **Alternative deep dehydrogenation pathways via CH_2CCH_3 intermediate…………...7**
4. **The CH_3 binding energy over Pt/Cu SAA NPs and Pt/Cu SAA (111) slab model…....8**
5. **Energy profiles for PDH over (211) and (100) of Pt and Pt/Cu SAA……………….....9**
6. **The first two dehydrogenation steps over Pt/Ag SAA, Pt/Cu SAA and Pt(111)….….10**
7. **XRD patterns of the catalysts before and after reduction………..…………….......11**
8. **H_2-TPR profiles………………………………………………………………….... .12**
9. **DRIFTS of CO adsorbed on the catalysts……………………………………….....13**
10. **Morphology of the reduced 0.1Pt/Al_2O_3………………………………………....14**
11. **Morphology of the reduced 0.1Pt10Cu/Al_2O_3…..……………………………. .15**
12. **HAADF-STEM images of the reduced 0.1Pt10Cu/Al_2O_3………………………16**
13. **HAADF-STEM images of the reduced 10Cu/Al_2O_3……………………………. .17**
14. **Pt L3 edge XANES……………………………………………………………...18**
15. **R space Pt L3 edge EXAFS……………………………………………………19**
16. **Cu K edge XANES…………………………………………….............………....22**
17. **R space Cu K edge EXAFS……………………………………………………23**
18. **TPSR of P-D scrambling and catalytic performances over Pt/Ag SAA……….26**
19. **Raman spectra of the spent catalysts…………………………………………27**
20. **TPD of C_3H_6………………………………………………………………………28**
21. **Catalytic performances after pre-reduction at different temperatures…….…29**
22. **Catalytic performances of 0.1Pt/Al_2O_3 and 0.1Pt10Cu/Al_2O_3…………………30**
23. **Catalytic performances of 0.1Pt10Cu/Al_2O_3 after five cycles at 520 ℃…………….31**
24. **Elemental mapping of 0.1Pt10Cu/Al_2O_3 after five cycles at 520 ℃…………...….32**
25. **Morphology of 0.1Pt10Cu/Al_2O_3 after five cycles at 520 °C………………………33**
26. **Catalytic performances of 0.1Pt10Cu/Al_2O_3 after four cycles at 600 ℃...………..…34**
27. **Elemental mapping of 0.1Pt10Cu/Al_2O_3 after four cycles at 600 ℃……...………35**
28. **Morphology of 0.1Pt10Cu/Al_2O_3 after four cycles at 600 °C………...……………36**
29. **Catalytic performances of 10Cu/Al_2O_3…………………………………………….37**

30. Morphology of the 0.1Pt10Cu/Al_2O_3 catalyst after 120 h reaction at 520 ℃……...…38

31. Morphology of 0.1Pt10Cu/Al_2O_3 before and after reaction at 600 ℃…...…………….39

32. C_3H_6 adsorption energy (eV) over Pt(111), Pt_3Cu(111) and Pt/Cu SAA………40

33. Relative stability (eV) of single atom Pt with Cu surfaces…………………………..….41

34. Relative stability (eV) of single atom Pt on Cu nanoparticles………………...…..…42

35. Relative stability (eV) of dimer Pt on Cu nanoparticles (> 2.1 nm)……………….……..43

36. Calculated top adsorbed CO frequency and experimentally measured one…………44

37. Relative stability (eV) of single atom Pt with Ag surfaces……………………….......45

38. XANES edge energies and EXAFS fitting results at the Pt L3 and Cu K…………..…46

39. Physicochemical parameters of 0.1Pt/Al_2O_3, 0.1Pt10Cu/Al_2O_3, and 10Cu/Al_2O_3…....47

40. Catalytic properties of PDH over 0.1Pt/Al_2O_3 and 0.1Pt10Cu/Al_2O_3…………………48

41. Catalytic properties of PDH over some representative Pt-based catalysts…………...49

42. References………………………………………………………………………....51

Supplementary Note | Methods for characterization

Textual properties of catalysts were measured with a Micromeritics Tristar 3000 analyzer by nitrogen adsorption at -196 ℃. Prior to measurements, the catalysts were outgassed at 200 ℃ for 3 h. The Brunauer-Emmett-Teller (BET) method was employed to determine the specific surface area (S_{BET}) by measuring the quantity of nitrogen adsorbed at -196 ℃.

Elemental composition of the catalysts was analyzed by inductively coupled plasma optical emission spectroscopy (ICP-OES) (VISTA-MPX, Varian). Before measurements, the catalysts were digested in the mixed solutions of H_3PO_4 and aqua regia.

The X-ray diffraction (XRD) measurements were performed on a Bruker D8 diffractometer operating at 200 mA and 40 kV, employing the graphite filtered Cu Kα as the radiation source. The data points were collected by step scanning with a rate of 6 ° min^{-1} from 2θ = 25 to 85 °. The crystallite size (d) of copper was calculated by X-ray broadening technique using the Scherrer's equation:

$$d = 0.89\lambda / (B \cdot \cos\theta) \quad (1)$$

Here, λ is the wavelength of the radiation source (0.15418 nm); B is the half width of the strongest diffraction peak in the radian unit; and θ is its diffraction angle.

Raman spectra were collected to investigate the coke deposited on the surface of the catalysts after propane dehydrogenation by a Renishaw inVia Reflex Raman spectrometer equipped with 532 nm Ar-ion laser beam.

H_2-O_2 titration method was used to determine the dispersion of Pt with a Micromeritics AutoChem II 2920 apparatus with a thermal conductivity detector based on our prior works.[1] For every test, 100 mg of sample was reduced at 600 ℃ with a flow rate of 30 mL min^{-1} of 10 vol% H_2/Ar for 1h, and then cooled down to 50 ℃ under Ar purging. Subsequently, 10 vol% O_2/He was introduced to the sample by injection pulses until the consumption peaks became stable. Finally, H_2 chemisorption was performed by injection pulses of 10 vol% H_2/Ar. It can be presumed that the adsorption stoichiometry factor of Pt/H_2 was equal to 2/3. The platinum dispersion is calculated by the following equation:

$$\text{Dispersion (\%)} = 100 \times V_{H2} \times 2/3 \times MW_{Pt} / (W_{Pt} \times 22414) \quad (2)$$

Where V_{H2} is the volume of adsorbed H_2 (mL), MW_{Pt} is the atomic weight of Pt (g mol^{-1}), and W_{Pt} is the weight of Pt supported on the sample (g).

Temperature-programmed experiments were all carried out with a Micromeritics AutoChem 2920 apparatus. For H_2-TPR tests, 100 mg of sample was heated at 300 ℃ for 1 h and cooled down to 50 ℃ in a flow of Ar (30 mL min^{-1}) and then reduced in a stream of 10 vol% H_2/Ar (30 mL min^{-1}) at a heating rate of 10 ℃ min^{-1} up to 800 ℃. For C_3H_6-TPD experiments, 100 mg of sample was first reduced in flowing 10 vol% H_2/Ar at 600 ℃ for 1h. Then, the sample was cooled down to 50 ℃ and the gas was switched to flowing Ar to flush the apparatus. Subsequently, the adsorption of C_3H_6 was achieved by the introduction of flowing C_3H_6 for 45 min, and then the system was purged with flowing He for 30 min. After that, the sample was raised at a rate of 10 ℃ min^{-1} up to 600 ℃ in flowing He. The exhaust from the reactor was analysed by a TCD detector.

Supplementary Note | Results of extended X-ray absorption fine structure (EXAFS)

Supplementary Table 1 shows the XANES edge energies and EXAFS fitting results for 0.1Pt/Al_2O_3 and 0.1Pt6.7Cu/Al_2O_3 catalysts at the Pt L3 edge. Pt L3 edge XANES spectra are shown in Supplementary Figure 12. For the 0.1Pt6.7Cu/Al_2O_3 catalyst, the XANES edge energy was above that of Pt foil, which can be attributed to both oxidation and alloy formation. The white line intensity of the bimetallic Pt-Cu catalyst was also higher than the foil, which is typical of oxidized platinum. Pt-Cu alloy formation typically makes the white line shorter and broader than platinum foil[2]. R space spectra of the bimetallic catalyst (Supplementary Figure 13) showed peaks typical of Pt-O and Pt-3d scattering. Fits of the first shell gave a Pt-O coordination of 1.4 at a bond distance of 2.04 angstroms and a Pt-Cu coordination of 4.4 at a bond distance of 2.53 angstroms. Platinum in the sample had no platinum nearest neighbours. First shell EXAFS R space fits at the Pt L3 edge of 0.1Pt6.7Cu/Al_2O_3 are shown in Supplementary Figure 14.

For the monometallic platinum catalyst, the XANES edge energy was 11564.4 eV, above that of the foil. Similar to the bimetallic sample, the white line intensity was above that of the foil. R space spectra showed peaks typical of Pt-O and Pt-Pt scattering. Fits of the first shell

gave a Pt-O coordination number of 1.6 at a bond distance of 2.03 angstroms and a Pt-Pt coordination number of 4.4 at a bond distance of 2.73 angstroms. Accounting for the fraction of the sample in the metallic state, the true Pt-Pt coordination number is 7.3, corresponding to a metal particle size of approximately 2.5 nanometers.

Cu K edge XANES spectra of 0.1Pt6.7Cu/Al_2O_3 after hydrogen and propane treatments are shown in Supplementary Figure S16. The XANES edge energy of both the hydrogen treated and propane treated bimetallic Pt-Cu catalyst matched the foil value of 8979 eV, which is consistent with metallic copper nanoparticles. The R space spectra at the copper edge of the bimetallic catalysts (Supplementary Figure 17) show a single Cu-Cu scattering peak in the first shell, and higher shell scattering lower in intensity but identical in shape to that of the foil. The hydrogen treated sample (Supplementary Figure 18) had a Cu-Cu coordination number of 7 at a bond distance of 2.55 angstroms. After treatment with propane, the Cu-Cu coordination number increased to 8.4 and the bond distance changed to 2.56 angstroms (fit shown in Supplementary Figure 19). The change is consistent with sintering of the Cu particles in the sample due to the high temperature treatment.

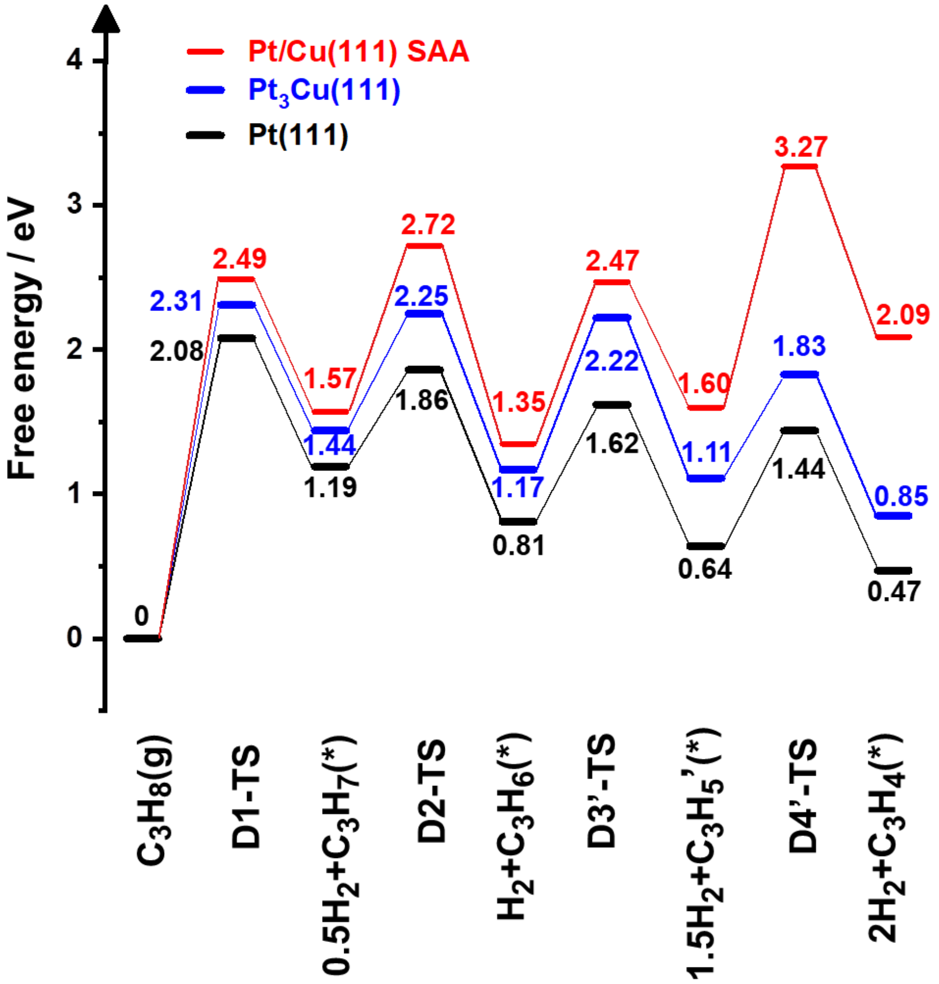

Supplementary Figure 1 | Alternative deep dehydrogenation pathways via CH_2CCH_3 intermediate. The first two dehydrogenation steps are the same as shown in Fig. 1c in the main text.

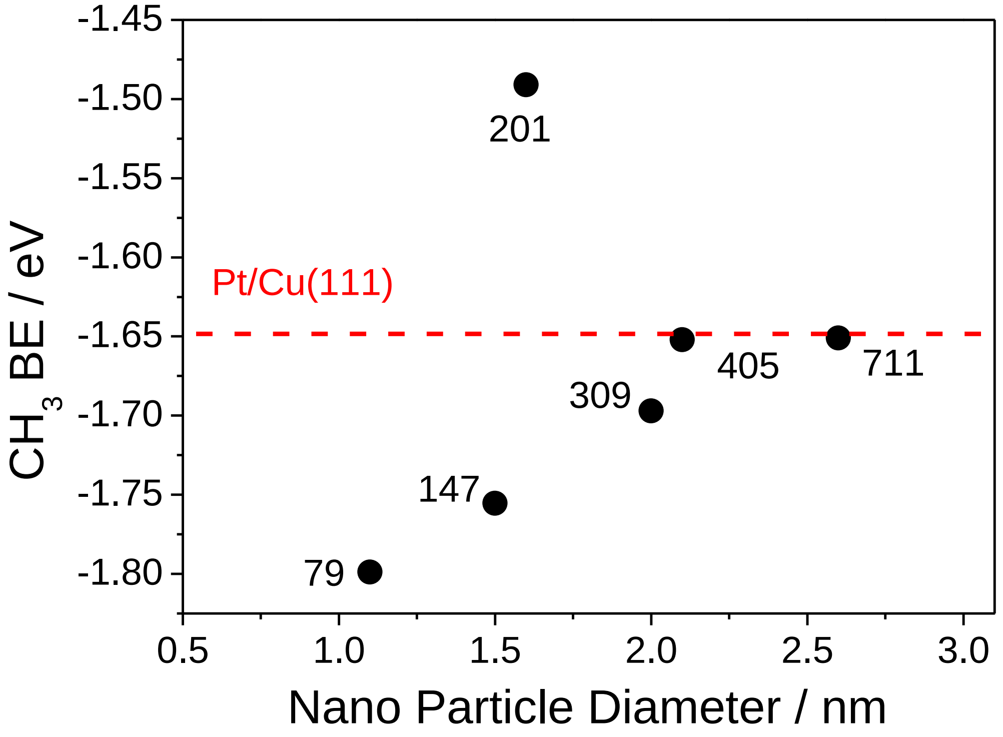

Supplementary Figure 2 | The calculated CH_3 binding energy over Pt/Cu SAA nanoparticles and Pt/Cu SAA (111) slab model. The number in the figure indicates the total number of metal atoms of the model cuboctahedron nanoparticle.

The Convergence test was done with nanoparticles modelled by truncated octahedra and cuboctahedron exposing (111), (100) surfaces and (211) like edges. One Cu atom, located at the middle of (111) facets, was replaced by a Pt atom and the CH_3 was binding on top site of this Pt atom. Supplementary Figure 2 shows that the CH_3 binding energy coverages when the nanoparticle diameter is larger than 2 nm (Pt/Cu_{404}), indicating our (111) slab model could well represent Pt/Cu SAA nanoparticles which are larger than 2 nm.

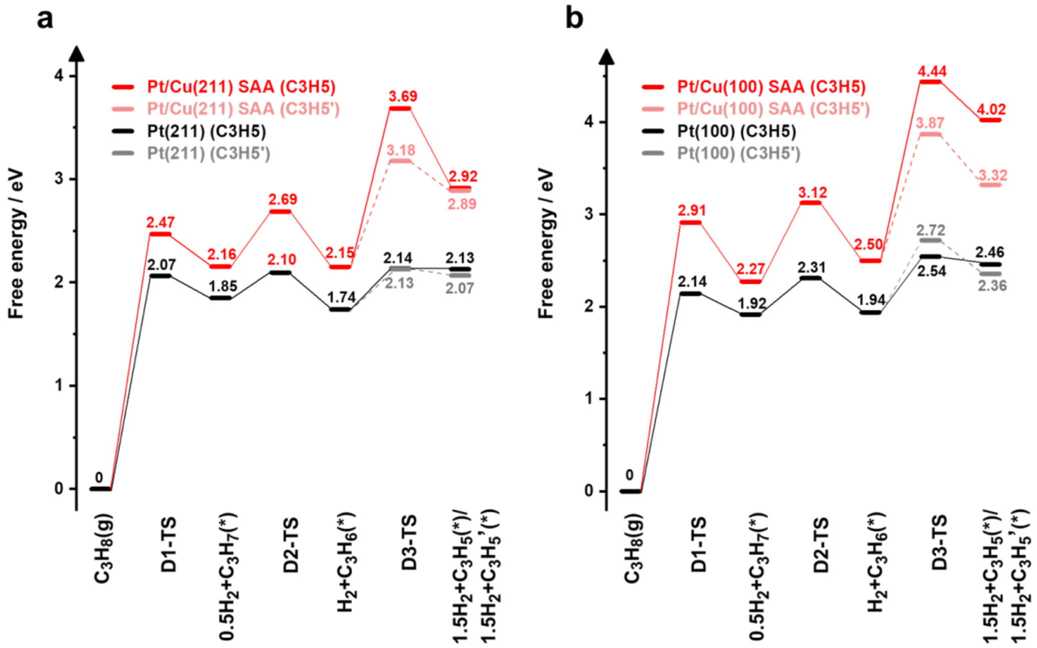

Supplementary Figure 3 | Energy profile for propane dehydrogenation over (a) Pt(211) and Pt/Cu(211) SAA; (b) Pt(100) and Pt/Cu(100) SAA. For both Pt/Cu SAA surfaces, a dramatic increase of the propylene dehydrogenation barrier has been observed, indicating their coke resistance ability similar as Pt/Cu(111) SAA. However, the Pt/Cu(100) might have a low PDH activity due to its relative high propane dehydrogenation barrier.

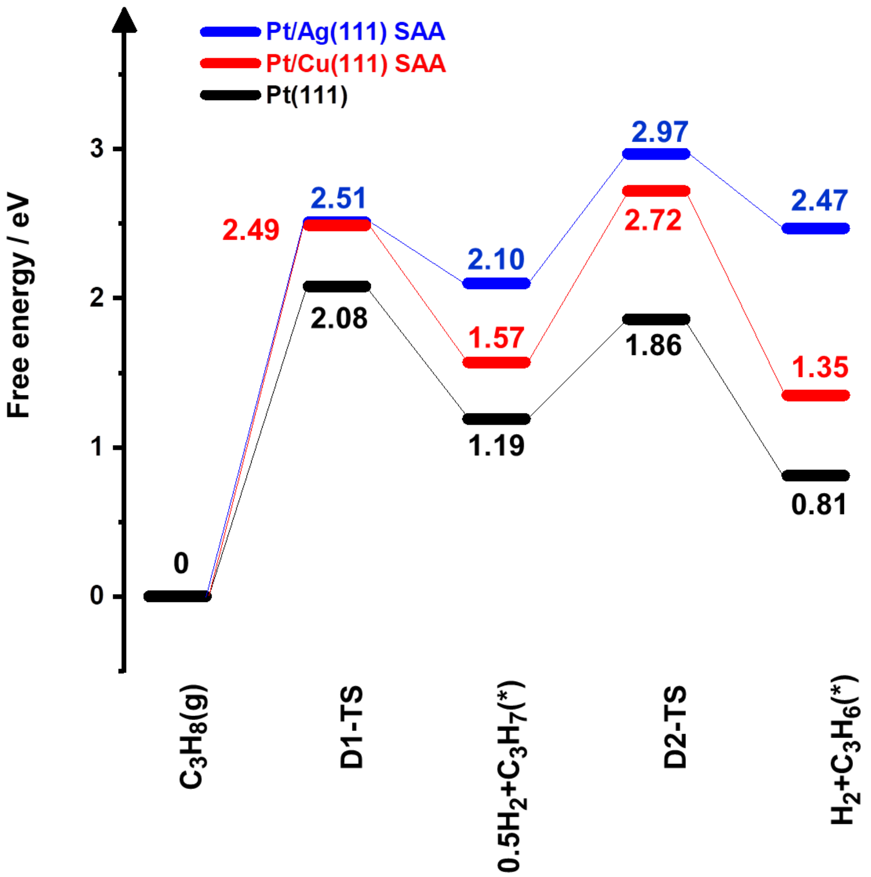

Supplementary Figure 4 | Comparison of the first two dehydrogenation steps over Pt/Ag SAA, Pt/Cu SAA and Pt(111). Energy profiles of the first two dehydrogenation steps over Pt/Ag SAA, Pt/Cu SAA and Pt(111).

Supplementary Figure 4 shows very close reaction barriers of the first two dehydrogenation steps over Pt/Ag SAA and Pt/Cu SAA, demonstrating that Pt/Ag SAA has similar intrinsic activity with Pt/Cu SAA. According to the results of TPSR of P-D scrambling (Supplementary Figure 20a), the C-H activation starts at about 188 ℃ on Pt nanoparticles, 204 ℃ on Pt/Cu SAA, and 214 ℃ on Pt/Ag SAA, which confirms single atoms of Pt dispersed on Ag and Cu nanoparticles have a comparable intrinsic activity with Pt nanoparticles. Although the conversion of propane over Pt/Ag SAA under reaction conditions was lower than that over Pt/Cu SAA (Supplementary Figure 20b), this can be attributed to the higher stability for single atoms of Pt on the subsurface than on the surface of Ag nanoparticles, leading to less exposed Pt active sites (Supplementary Table 6). The results of DFT calculations and TPSR of P-D scrambling experiments suggest a promotion of breaking scaling relationship by forming single Pt atoms on metal nanoparticles.

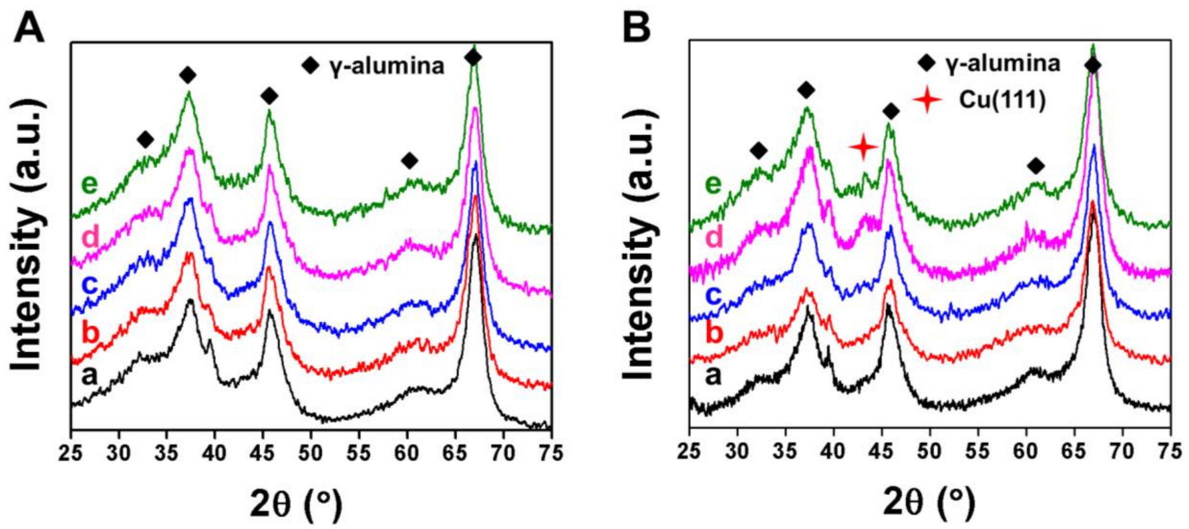

Supplementary Figure 5 | XRD patterns of the catalysts before and after reduction. XRD patterns of (a) 0.1Pt/Al_2O_3, (b) 0.1Pt3Cu/Al_2O_3, (c) 0.1Pt5Cu/Al_2O_3, (d) 0.1Pt6.7Cu/Al_2O_3, and (e) 0.1Pt10Cu/Al_2O_3 after calcination at 600 ℃ for 2 h (A) and after reduction at 600 ℃ for 1 h (B).

Supplementary Figure 5A shows the XRD patterns of the catalysts after calcination at 600 ℃. Only diffraction lines due to γ-alumina (JCPDS 10-0425) were detected, suggesting that Cu oxide species exist as highly dispersed species on the surface of alumina. The XRD patterns of the catalysts after reduction at 600 ℃ are shown in Supplementary Figure 5B. The diffraction line of Cu (111) becomes apparent as the content of Cu increases to 6.7 wt%, demonstrating the formation of Cu nanoparticles on γ-alumina. Besides, the average crystalline size `of Cu derived from diffraction line corresponding to Cu (111) at 43.3° are 3.4 nm and 4.1 nm for 0.1Pt6.7Cu/Al_2O_3 and 0.1Pt10Cu/Al_2O_3. Note that no diffraction lines assigned as Pt species were detected due to its extremely low loading.

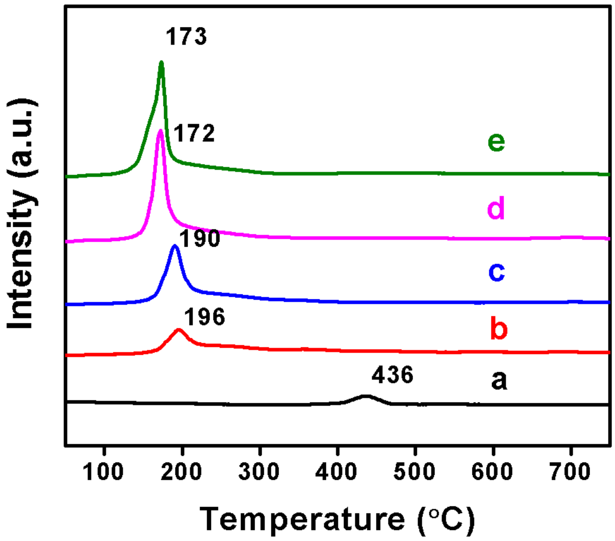

Supplementary Figure 6 | H_2-TPR profiles. H_2-TPR profiles of (a) 0.1Pt/Al_2O_3, (b) 0.1Pt3Cu/Al_2O_3, (c) 0.1Pt5Cu/Al_2O_3, (d) 0.1Pt6.7Cu/Al_2O_3, and (e) 0.1Pt10Cu/Al_2O_3 after calcination at 600 ℃ for 2 h.

Supplementary Figure 6 shows the H_2-TPR profiles of the catalysts with different content of Cu. The monometallic Pt catalyst exhibits one high-temperature reduction peak at around 436 ℃, which is assigned as the reduction of Pt oxide species strongly interacting with γ-alumina. Compared with 0.1Pt/Al_2O_3, the bimetallic PtCu catalysts presents one low-temperature reduction peak due to co-reduction of highly dispersed Cu oxide species and Pt oxide species. Moreover, the peak temperature decreases from 196 ℃ to 172 ℃ as the content of Cu increases from 3 wt% to 10 wt%, which is fairly consistent with the previous study.[3] Considering that the appearance of the co-reduction peaks means the close contact between Cu oxide species and Pt oxide species, it seems that the formation of PtCu bimetallic nanoparticles is preferred rather than two metal phases with Pt and Cu apart from each other during reduction. When treated under H_2-rich condition at 600 ℃ for 1h, the oxide species of Pt and Cu on γ-alumina can be fully reduced since the highest reduction peak temperature for PtCu bimetallic catalysts is below 200 ℃.

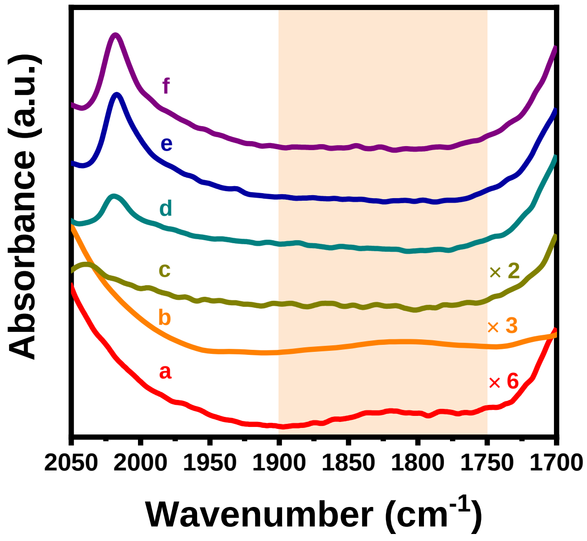

Supplementary Figure 7 | DRIFTS of CO adsorbed on the catalysts. DRIFTS of bridge-bond CO adsorbed on (a) 0.1Pt/Al_2O_3, (b) 0.1Pt0.1Cu/Al_2O_3, (c) 0.1Pt0.3Cu/Al_2O_3, (d) 0.1Pt3Cu/Al_2O_3, (e) 0.1Pt6.7Cu/Al_2O_3, and (f) 0.1Pt10Cu/Al_2O_3.

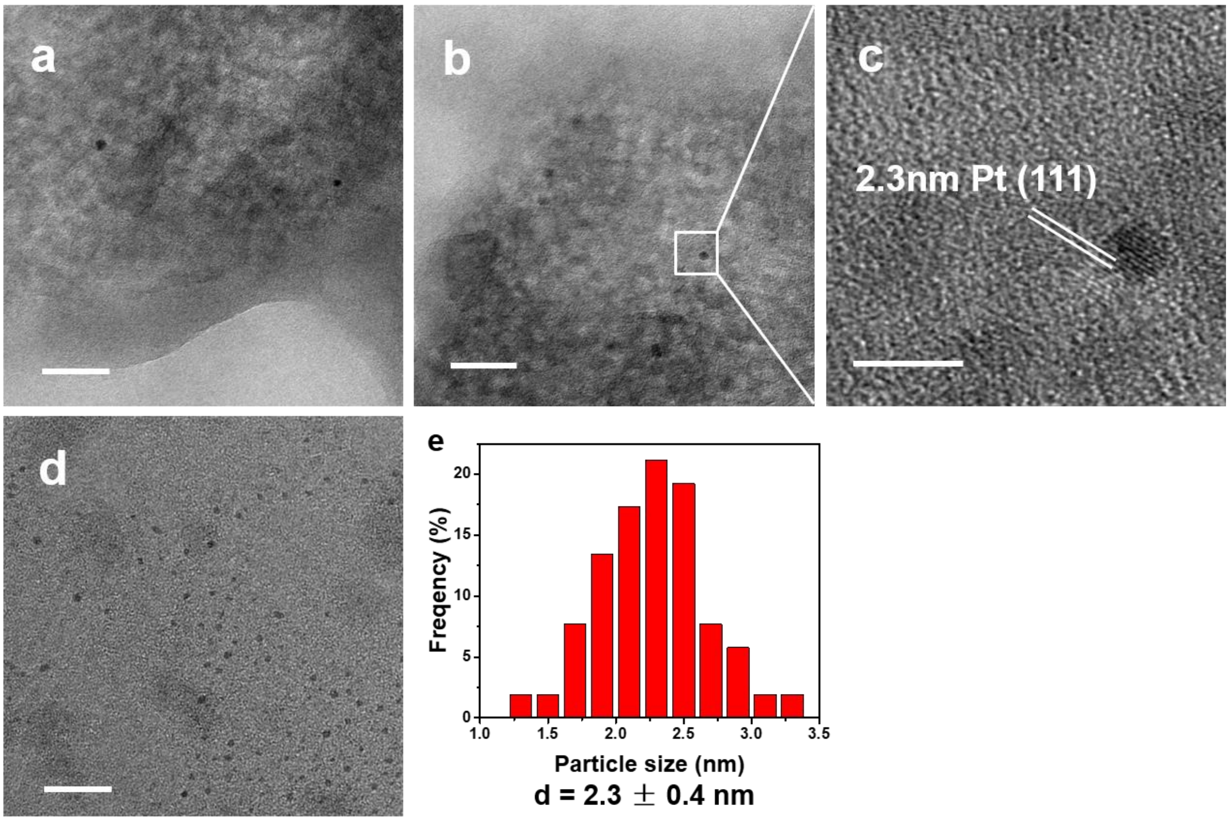

Supplementary Figure 8 | Morphology of the reduced 0.1Pt/Al_2O_3. TEM images of 0.1Pt/Al_2O_3 after reduction in the flow of 18 vol% H_2/N_2 at 600 ℃ for 1h. Scale bars, 20 nm (a), (b), (d) and 5 nm (c).

Typical TEM images of the reduced 0.1Pt/Al_2O_3 catalyst are shown in Supplementary Figure 8. The lattice spacing of Pt facets in Supplementary Figure 8c is consistent with that of metallic Pt (111). Moreover, the mean diameter of Pt nanoparticles was estimated to be about 2.3 nm.

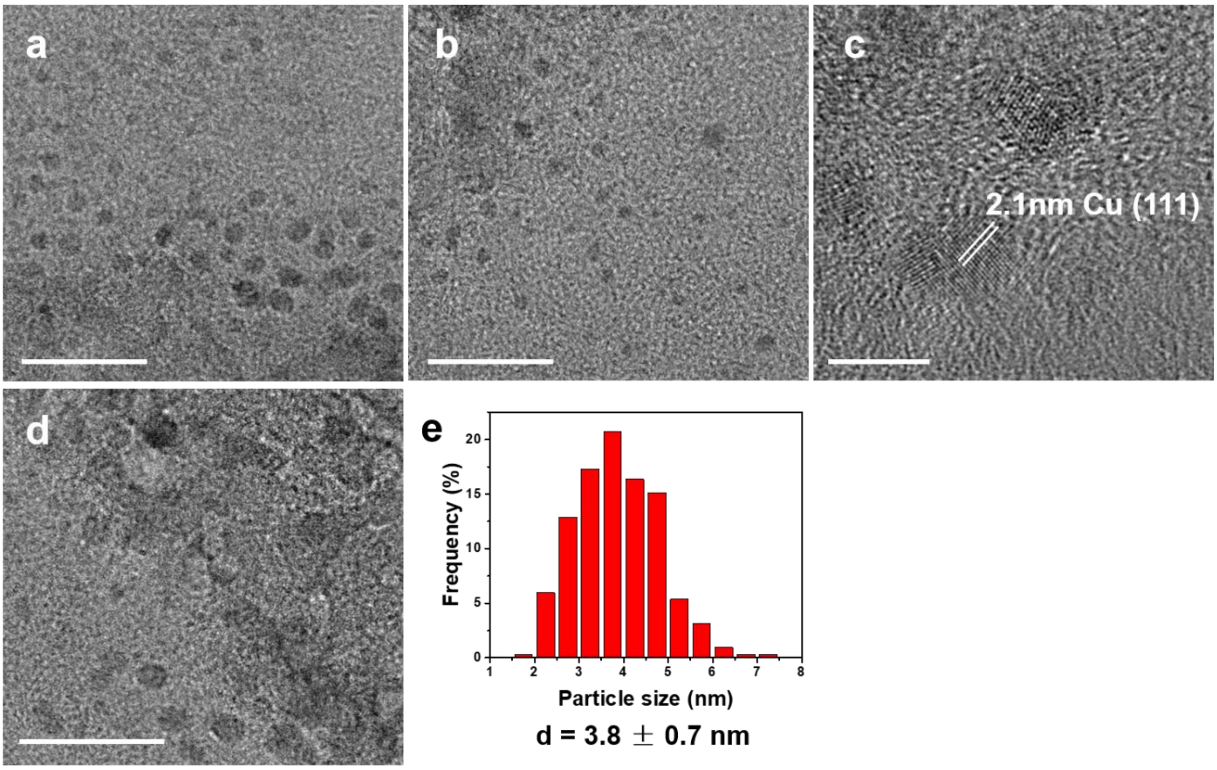

Supplementary Figure 9 | Morphology of the reduced 0.1Pt10Cu/Al_2O_3. TEM images of 0.1Pt10Cu/Al_2O_3 after reduction in the flow of 18 vol% H_2/N_2 at 600 ℃ for 1h. Scale bars, 20 nm (a), (b), (c) and 5 nm (d).

Typical images of the reduced 0.1Pt10Cu/Al_2O_3 catalyst are shown in Supplementary Figure 9. The lattice spacing of Cu facets in Supplementary Figure 9c is in line with that of metallic Cu (111), which suggests that the addition of 0.1 wt% Pt did not change the lattice structure of Cu. The average crystalline size of 0.1Pt10Cu/Al_2O_3 was found to be 3.8 nm, which is similar to that based on FWHM of Cu (111) as determined by the Scherrer equation (Supplementary Figure 5B).

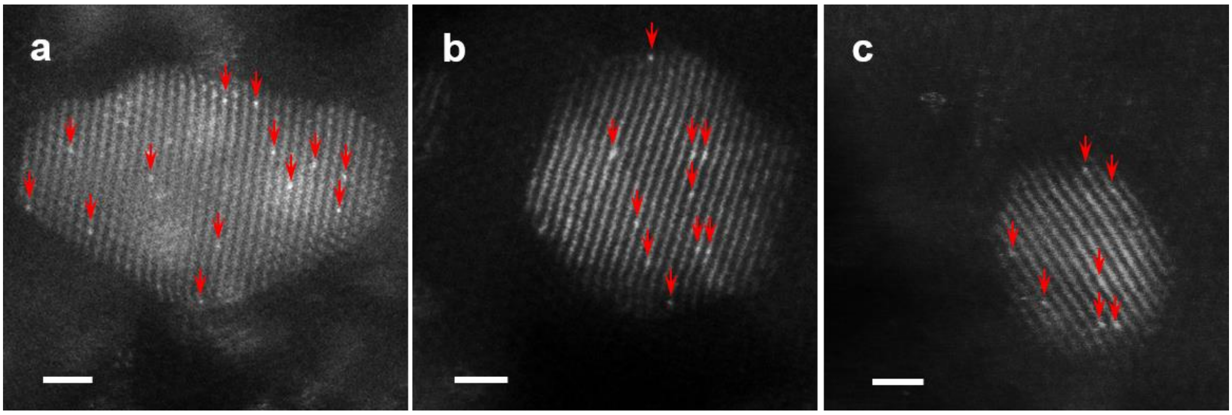

Supplementary Figure 10 | Morphology of the reduced 0.1Pt10Cu/Al_2O_3. Typical HAADF-STEM images of 0.1Pt10Cu/Al_2O_3 after reduction in the flow of 18 vol% H_2/N_2 at 700 ℃ for 1h, showing single atoms of Pt dispersed on Cu nanoparticles and no formation of Pt clusters or Pt nanoparticles. Single atoms of Pt are highlighted by red arrows. Scale bars, 1 nm (a), (b) and (c).

The reduced 0.1Pt10Cu/Al_2O_3 catalyst is clearly imaged by the AC-HAADF-STEM. The single Pt atoms can be identified from their higher brightness comparing to their surrounding area since Pt atoms are brighter than Cu atoms in the dark field STEM images. As shown vividly in Supplementary Figure 10, mainly single atoms of Pt are dispersed on Cu (111). The Pt atoms are highlighted by the red arrows. In contrast, there are no bright Pt atoms exist on Cu nanoparticles in the reduced 10Cu/Al_2O_3 catalyst (Supplementary Figure 11). Additionally, no fine clusters of Pt on γ-alumina were observed. It is worth noting that it is easy for Cu nanoparticles to melt when irradiated by high energy electron beams. The reduction temperature of 0.1Pt10Cu/Al_2O_3 for AC-HAADF-STEM was raised to 700 ℃ to obtain a relatively large Cu nanoparticle so as to withstand radiation. Note that the catalyst of 0.1Pt10Cu/Al_2O_3 has the same catalytic performance after reduction at 600 ℃ and 700 ℃, which means that the structure of the catalyst have no change when reduced at the two reduction temperatures (Supplementary Figure 23).

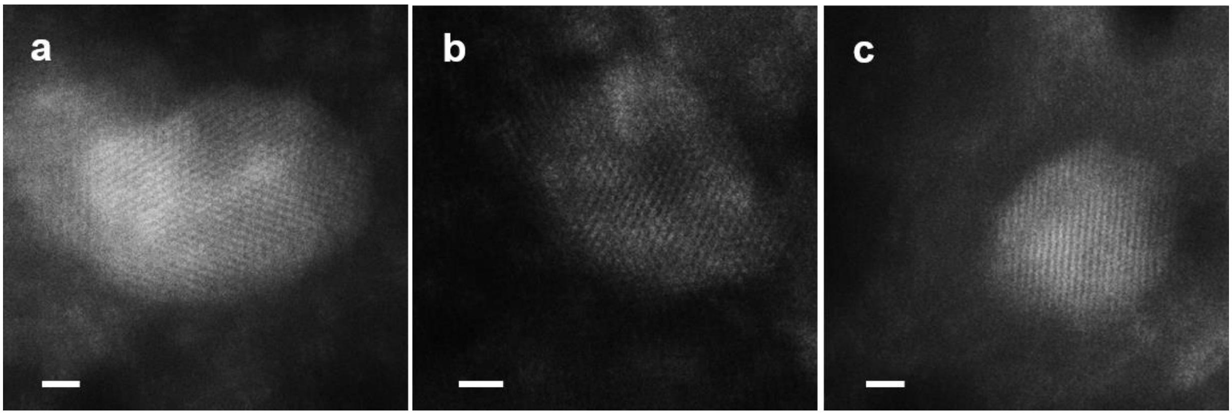

Supplementary Figure 11 | Morphology of the reduced 10Cu/Al_2O_3. HAADF-STEM images with typical regions of 10Cu/Al_2O_3 after reduction in the flow of 18 vol% H_2/N_2 at 700 ℃ for 1h. Scale bars, 1 nm (a), (b) and (c).

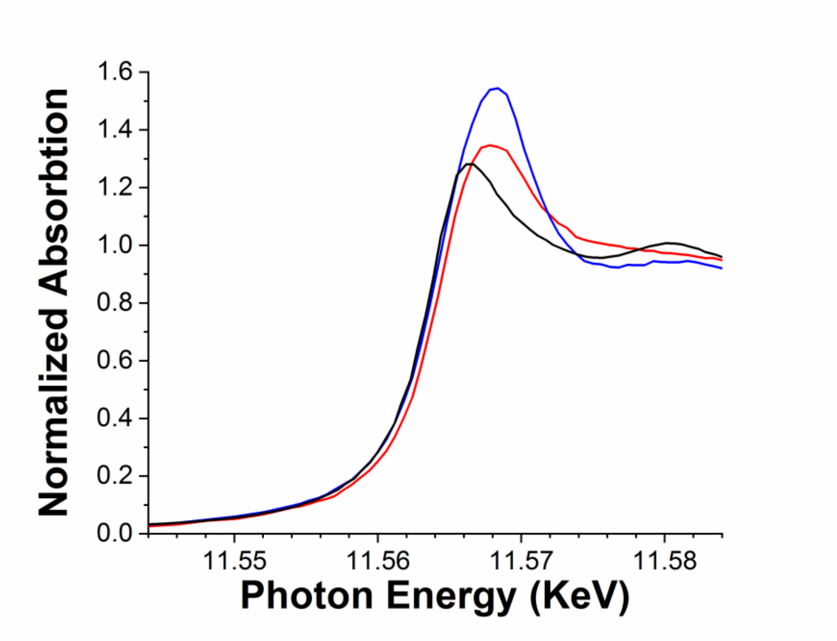

Supplementary Figure 12 | Pt L3 edge XANES of Pt foil (black), 0.1Pt/Al_2O_3 (blue) and 0.1Pt6.7Cu/Al_2O_3 (red). Spectra were recorded at 100 ℃ in 3% H_2 after a 30 min reduction in 3% H_2 at 550 ℃.

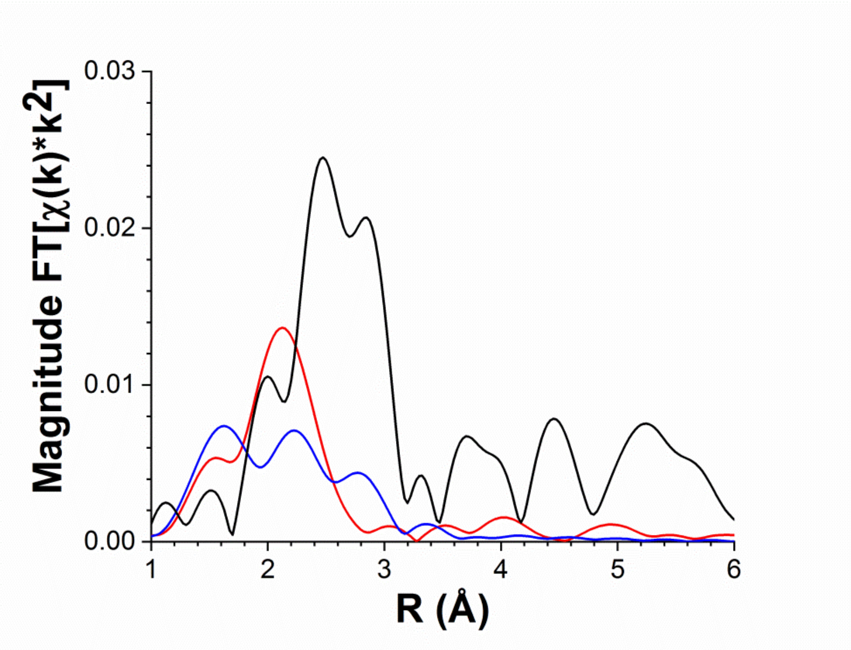

Supplementary Figure 13 | R space Pt L3 edge EXAFS of Pt foil (black), 0.1Pt/Al_2O_3 (blue) and 0.1Pt6.7Cu/Al_2O_3 (red). Spectra were recorded at 100 ℃ in 3% H_2 after a 30 min reduction at 550 ℃ in 3% H_2.

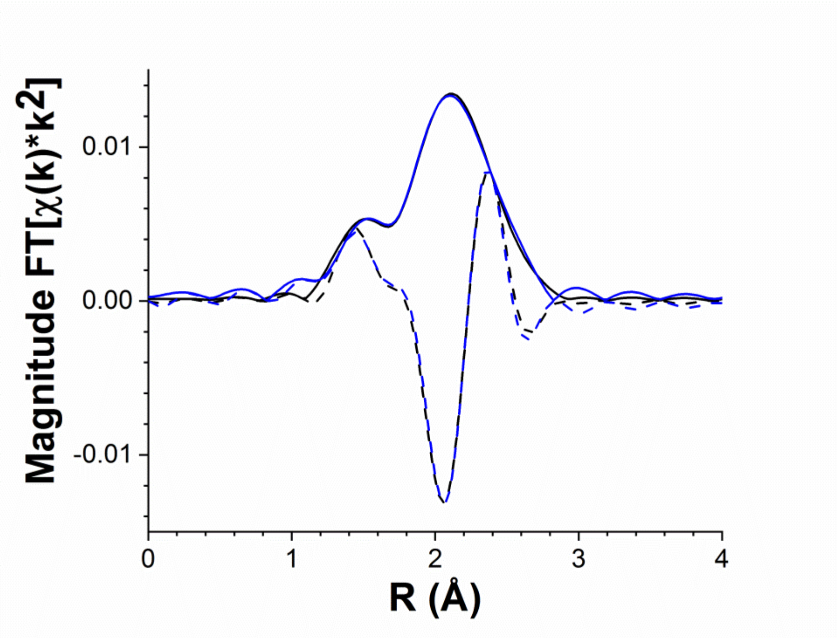

Supplementary Figure 14 | R space Pt L3 edge EXAFS spectra of 0.1Pt6.7Cu/Al_2O_3 magnitude (solid black) and imaginary (dashed black) component and R space fit of the EXAFS magnitude (solid blue) and imaginary (dashed blue) components.

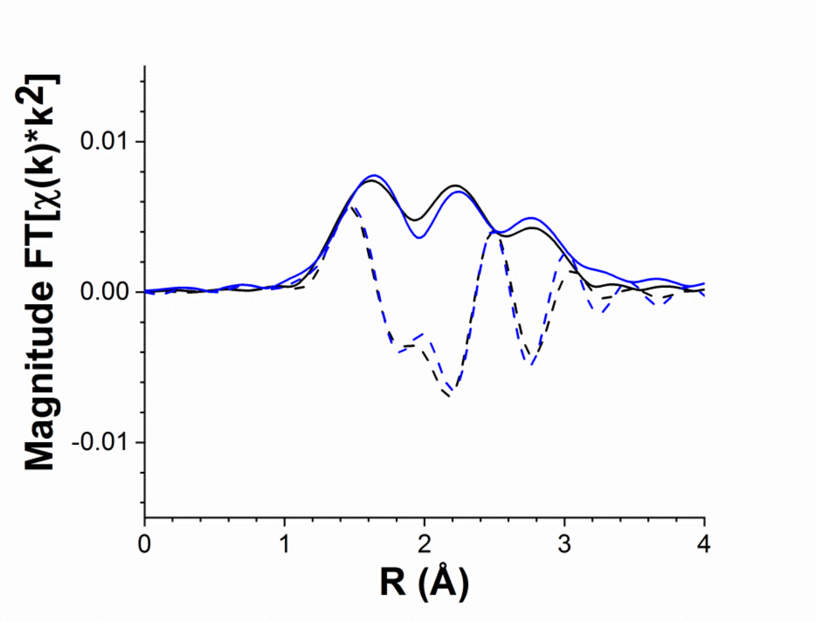

Supplementary Figure 15 | R space Pt L3 edge EXAFS spectra of 0.1Pt/Al_2O_3 magnitude (solid black) and imaginary (dashed black) component and R space fit of the EXAFS magnitude (solid blue) and imaginary (dashed blue) components.

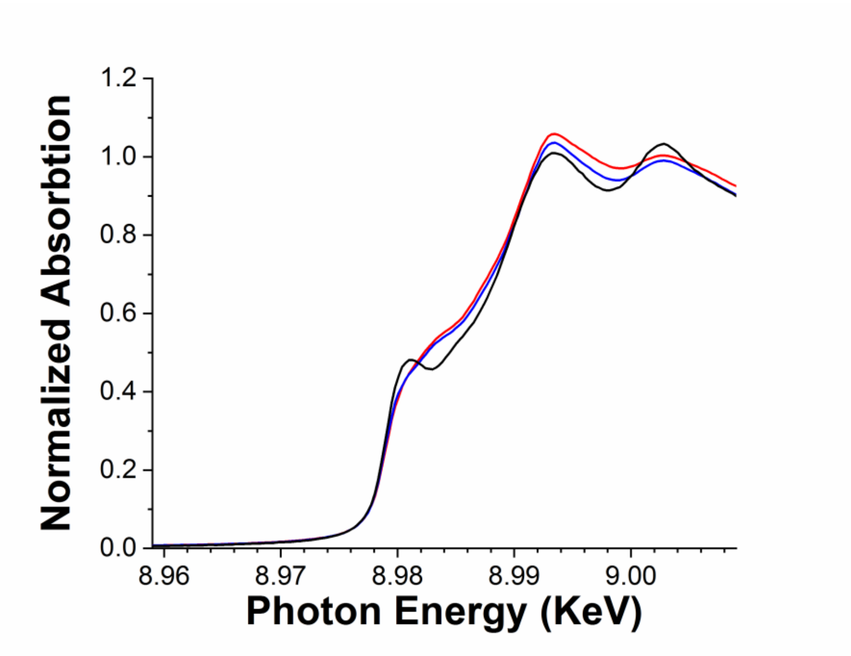

Supplementary Figure 16 | Cu K edge XANES of Cu foil (black) and 0.1Pt6.7Cu/Al_2O_3 after treatment in 3% H_2 (red) and 1% C_3H_8 (blue). Spectra were taken at room temperature, the hydrogen treated sample was measured in He, while the propane treated sample was measured in 1% propane.

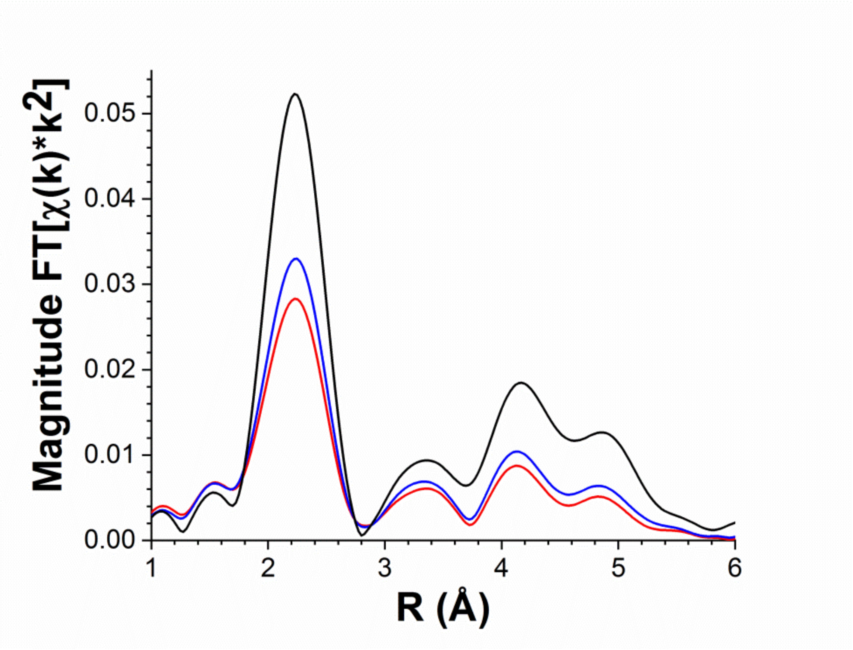

Supplementary Figure 17 | R space Cu K edge EXAFS spectra of Cu foil (black) and 0.1Pt6.7Cu/Al_2O_3 after treatment in 3% H_2 (red) and 1% C_3H_8 (blue). Spectra were taken at room temperature, the hydrogen treated sample was measured in He, while the propane treated sample was measured in 1% propane.

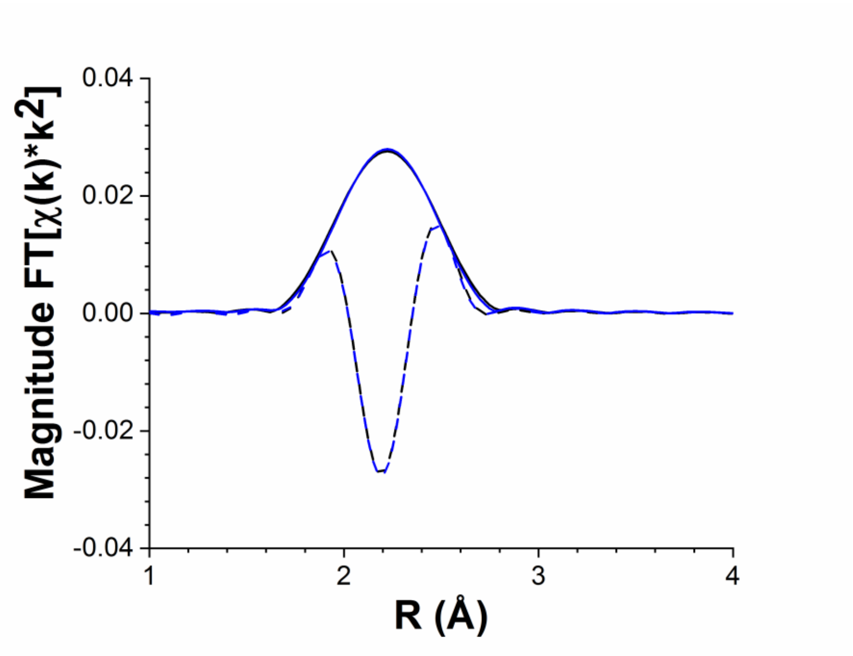

Supplementary Figure 18 | R space Cu L3 edge EXAFS spectra of Hydrogen treated 0.1Pt6.7Cu/Al_2O_3 magnitude (solid black) and imaginary (dashed black) component and R space fit of the EXAFS magnitude (solid blue) and imaginary (dashed blue) components.

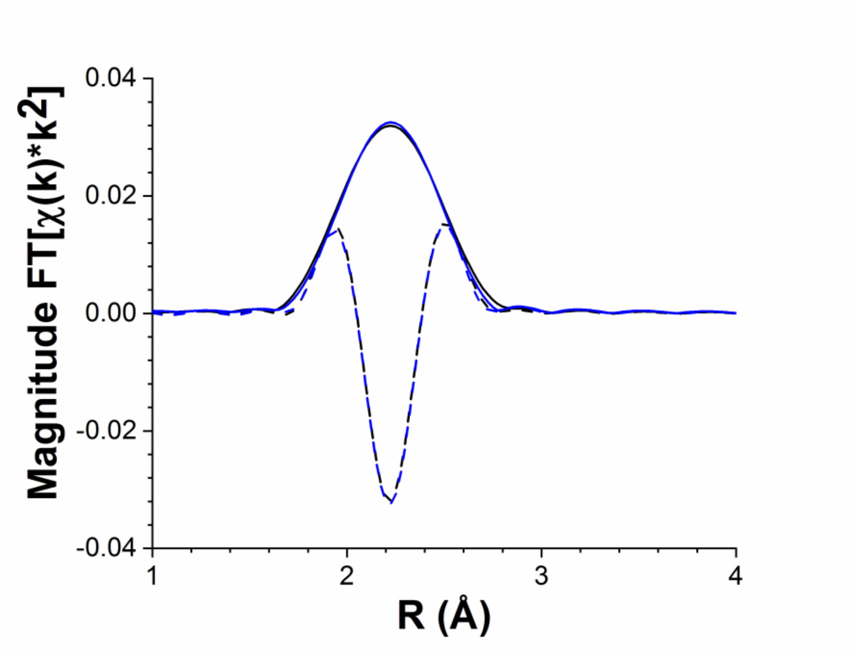

Supplementary Figure 19 | R space Cu L3 edge EXAFS spectra of propane treated 0.1Pt6.7Cu/Al_2O_3 magnitude (solid black) and imaginary (dashed black) component and R space fit of the EXAFS magnitude (solid blue) and imaginary (dashed blue) components.

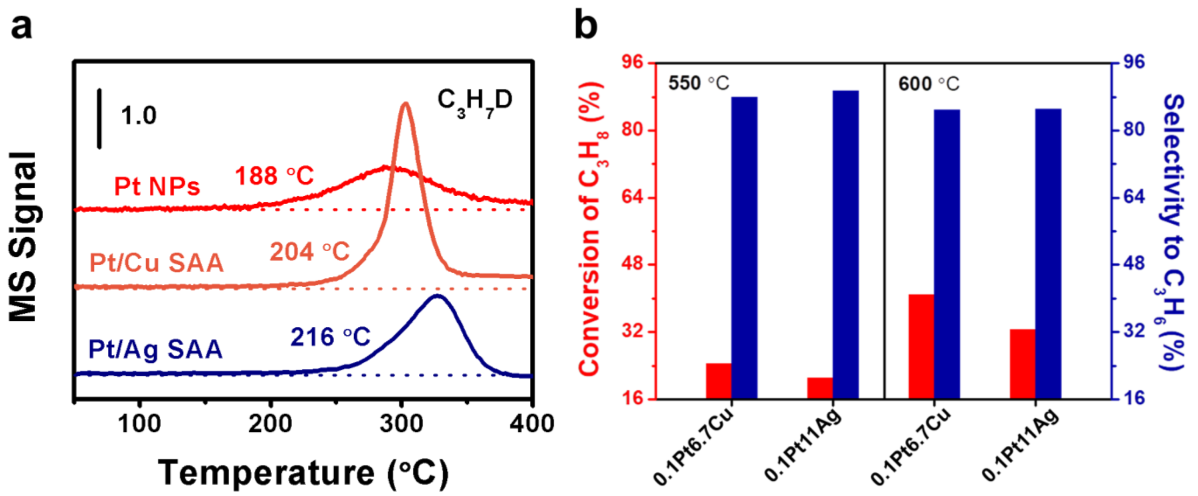

Supplementary Figure 20 | Comparison of the ability to activate C-H bonds and the catalytic performances of the catalysts. (a) Signals of C_3H_7D during TPSR over $0.3Pt/Al_2O_3$, $0.1Pt6.7Cu/Al_2O_3$ and $0.1Pt11Ag/Al_2O_3$ (atom ratio of Pt/M = 1/200, M = Ag and Cu) for propane-deuterium isotope scrambling (P-D scrambling). The mass ratio of 45/44 represents the level of C_3H_7D. (b) The catalytic performances of $0.1Pt6.7Cu/Al_2O_3$ and $0.1Pt11Ag/Al_2O_3$ at the initial period of propane dehydrogenation. Catalytic test conditions: atmospheric pressure, 550 ℃ and 600 ℃, WHSV of propane = 4 h^{-1}, 250 mg of sample, C_3H_8/H_2 = 1/1, with balance N_2 for total flow rate of 50 mL min^{-1}.

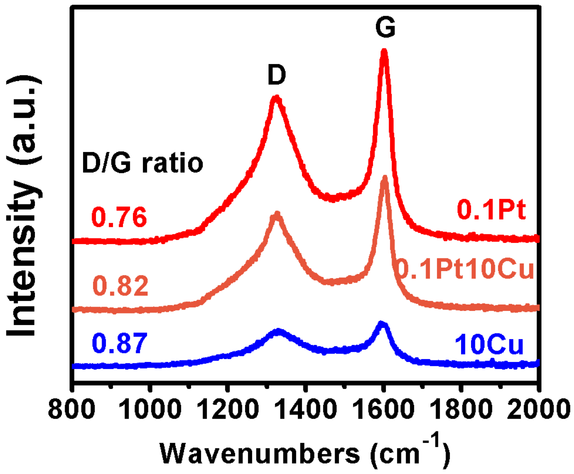

Supplementary Figure 21 | Raman spectra for the spent catalysts. Raman spectra of the catalysts of 0.1Pt/Al_2O_3, 0.1Pt10Cu/Al_2O_3 and 10Cu/Al_2O_3 after 12 hours on stream propane dehydrogenation. Catalytic test conditions: atmospheric pressure, 520 ℃, WHSV of propane = 4 h^{-1}, 250 mg of sample, C_3H_8/H_2 = 1/1, with balance N_2 for total flow rate of 50 mL min^{-1}.

Raman spectra of the spent catalysts were obtained to examine the degree of graphitization of the coke. The peaks at around 1326 and 1598 cm^1 were marked as D and G and the peak intensity ratio of D band to G band is usually used as an important index of the degree of graphitization of carbon materials[18]. The I_D/I_G values of 0.1Pt/Al_2O_3, 0.1Pt10Cu/Al_2O_3 and 10Cu/Al_2O_3 are 0.76, 0.82 and 0.87, repectively.

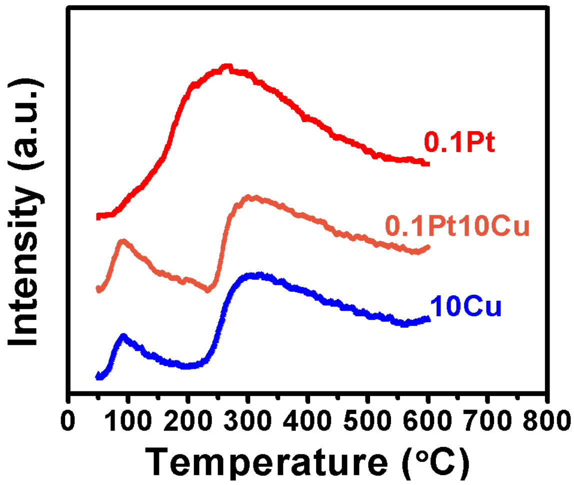

Supplementary Figure 22 | The desorption capacity of C_3H_6 over the catalysts. TPD of C_3H_6 adsorbed on the reduced catalysts of 0.1Pt/Al_2O_3, 0.1Pt10Cu/Al_2O_3 and 10Cu/Al_2O_3.

Supplementary Figure 22 shows one broad desorption peak of C_3H_6 on 0.1Pt/Al_2O_3 centred at about 280 ℃. For 0.1Pt10Cu/Al_2O_3 and 10Cu/Al_2O_3, two separated desorption peaks of C_3H_6 appear. The small low-temperature peak at around 90 ℃ can be attributed to desorption of C_3H_6 adsorbed on metal. While the main high-temperature peak centred at about 300 ℃ can be assigned as desorption of C_3H_6 on Al_2O_3. The C_3H_6 desorption property of 0.1Pt10Cu/Al_2O_3 is as similar as that of 10Cu/Al_2O_3.

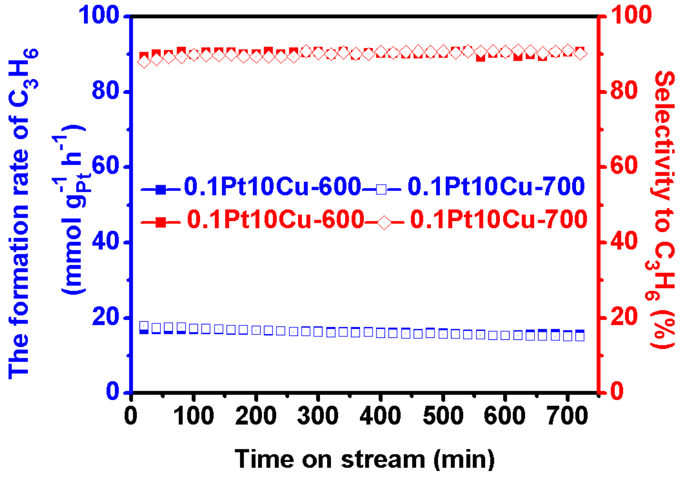

Supplementary Figure 23 | Comparison of the catalytic performances of 0.1Pt10Cu/Al_2O_3 after reduction at 600 ℃ and 700 ℃. Catalytic test conditions: atmospheric pressure, 550 ℃, WHSV of propane = 4 h^{-1}, 250 mg of sample, C_3H_8/H_2 = 1/1, with balance N_2 for total flow rate of 50 mL min^{-1}.

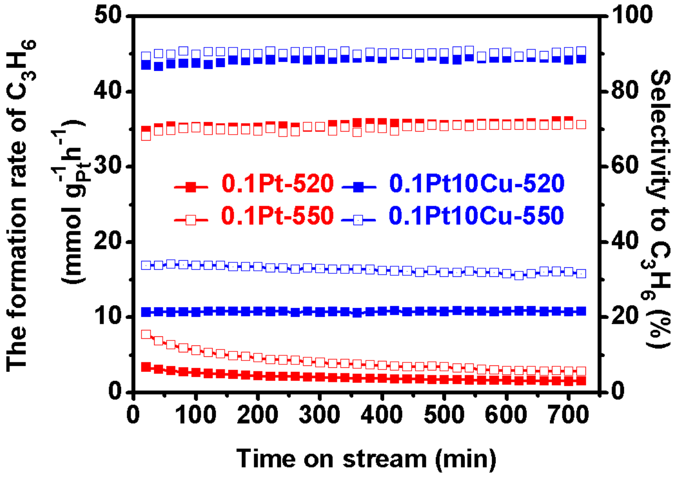

Supplementary Figure 24 | Comparison of the catalytic performances of 0.1Pt/Al_2O_3 and 0.1Pt10Cu/Al_2O_3. Catalytic test conditions: atmospheric pressure, 520 ℃ and 550 ℃, WHSV of propane = 4 h^{-1}, 250 mg of sample, C_3H_8/H_2 = 1/1, with balance N_2 for total flow rate of 50 mL min^{-1}.

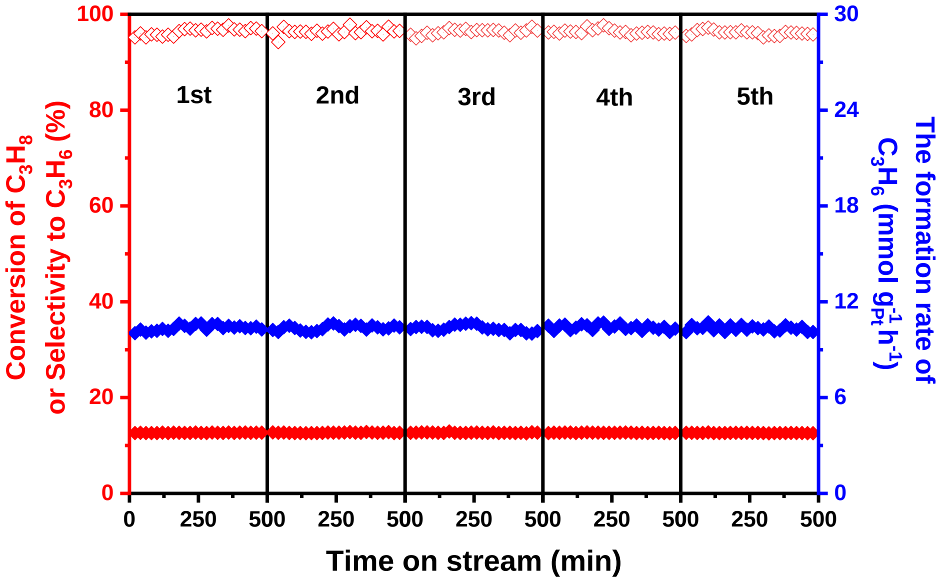

Supplementary Figure 25 | Catalytic performances on the 0.1Pt10Cu/Al_2O_3 catalyst for each of the five successive dehydrogenation cycles. Each cycle consists of a 8 h propane dehydrogenation step at 520 ℃, followed by a treatment in air at 520 ℃ for 45 min. The reactor is flushed with N_2 between these steps. Catalytic test conditions: atmospheric pressure, 520 ℃, WHSV of propane = 4 h^{-1}, 250 mg of sample, C_3H_8/H_2 = 1/1, with balance N_2 for total flow rate of 50 mL min^{-1}.

Five succesive dehydrogenation-regeneration cycles for the 0.1Pt10Cu/Al_2O_3 catalyst was carried out. At the end of the fifth propane dehydrogenation cycle, the conversion and selectivity of the catalyst did not drop, verifing its good stablility at 520 ℃.

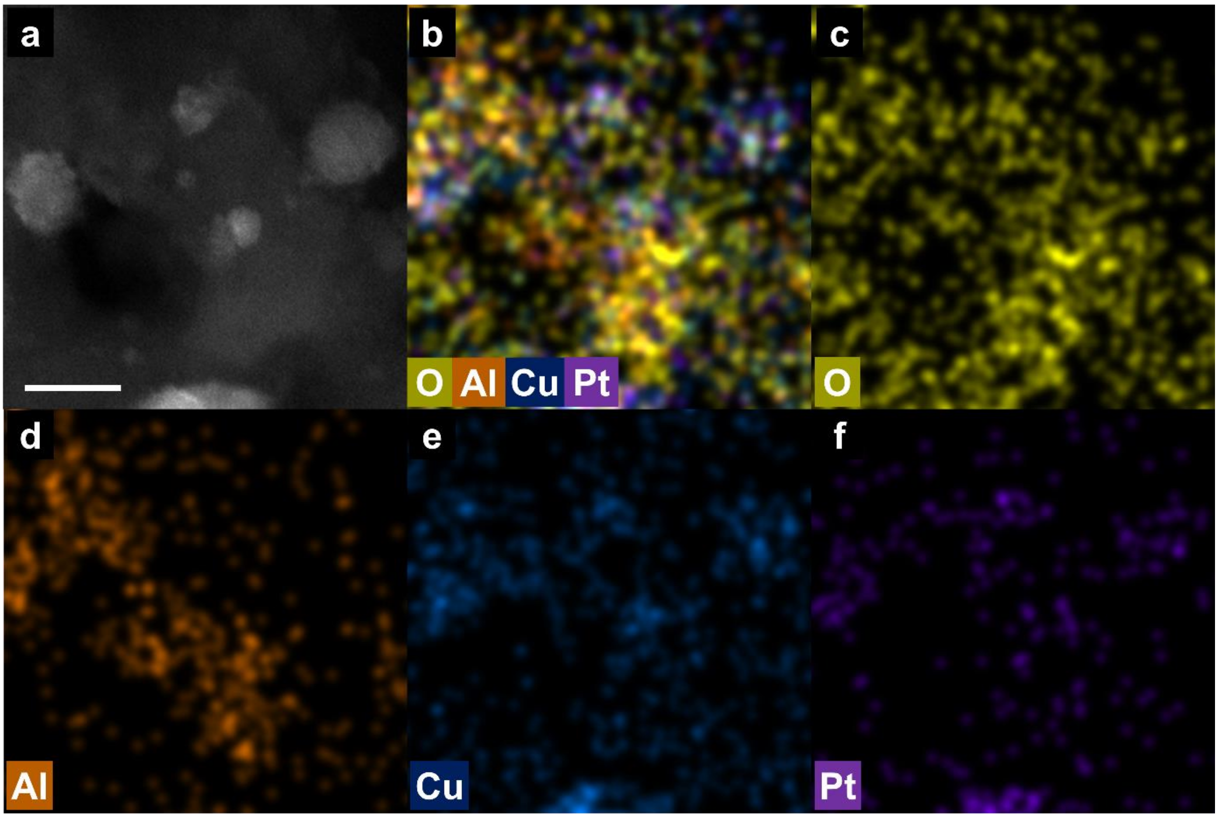

Supplementary Figure 26 | Elemental mapping of the 0.1Pt10Cu/Al_2O_3 catalyst after five successive dehydrogenation-regeneration cycles at 520 ℃. (a) HAADF STEM image of the 0.1Pt10Cu/Al_2O_3 catalyst. (b) EDS of the sample. (c) O, (d) Al, (e) Cu and (f) Pt elemental maps obtained from the same region on the sample. (b) EDS of the sample. Scale bar, 10 nm (a).

Supplementary Figure 26 shows the elemental maps by energy dispersive X-ray spectroscopy (EDS) of the 0.1Pt10Cu/Al_2O_3 catalyst, suggesting that Pt is mainly distributed on the Cu nanoparticles and not on the Al_2O_3 support by comparing the elemental maps of Pt and Cu.

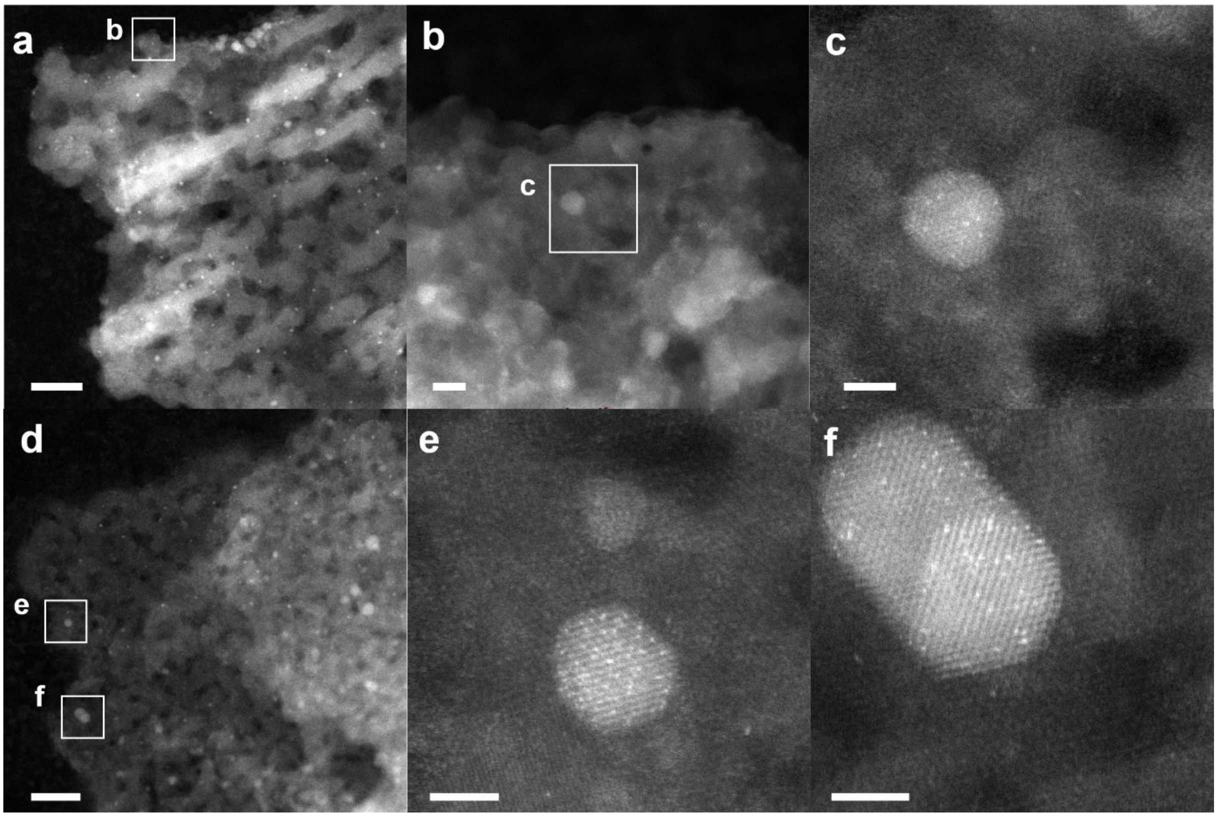

Supplementary Figure 27 | Morphology of the 0.1Pt10Cu/Al_2O_3 catalyst after five successive dehydrogenation-regeneration cycles at 520 ℃. (a) HAADF-STEM image with typical region of the 0.1Pt10Cu/Al_2O_3 catalyst. (b) The enlarged image from the selected region in (a). (c) The enlarged image from the selected region in (b), showing Pt atoms individually dispersed on Cu nanoparticle clearly. (d) HAADF-STEM image with typical region of the 0.1Pt10Cu/Al_2O_3 catalyst. (e,f) The enlarged images from the selected regions in (d), showing Pt atoms individually dispersed on Cu nanoparticles clearly. Scale bars, 20 nm (a) and (d), 5 nm (b), 2 nm (c), (e) and (f).

The 0.1Pt10Cu/Al_2O_3 catalyst after five cycles at 520 ℃ is clearly imaged by the AC-HAADF-STEM. Pt atoms identified from their higher brightness comparing to their surrounding area are individually dispersed on the Cu nanoparticles (Supplementary Figure 27), which is consistent with the state of Pt and Cu before oxidation-reduction cycles as shown in Figure 3 and Supplementary Figure 10.

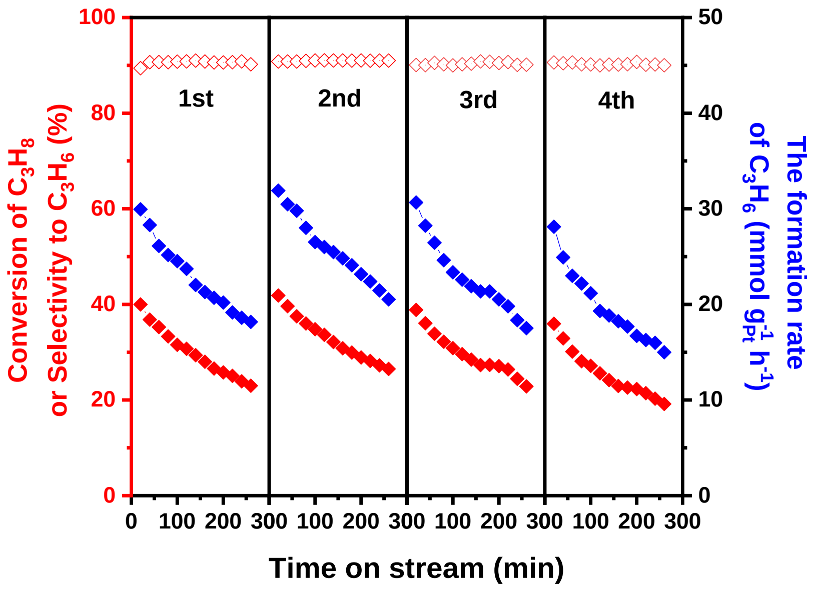

Supplementary Figure 28 | Catalytic performances on the 0.1Pt10Cu/Al_2O_3 catalyst for each of the four successive dehydrogenation cycles. Each cycle consists of a 4 h propane dehydrogenation step at 600 ℃, followed by a treatment in air at 600 ℃ for 45 min. The reactor is flushed with N_2 between these steps. Catalytic test conditions: atmospheric pressure, 600 ℃, WHSV of propane = 4 h^{-1}, 250 mg of sample, C_3H_8/H_2 = 1/1, with balance N_2 for total flow rate of 50 mL min^{-1}.

We raised the conversion level and carried out the catalytic test at 600 ℃, the catalyst deactivated quickly during each cycle and the initial activity of each cycle could be largely restored. The slightly drop of the initial activity after four cycles may be caused by the irreversible sintering of metal nanoparticles. As can be seen from Supplementary Figure 33, the sintering of PtCu SAA nanoparticles was noticed after reaction at 600 ℃ for 4 h. Although most of the sintered particles can be dispersed again after regeneration, there are still a few particles that were not well dispersed, leading to less exposed Pt atoms on the surface of Cu nanoparticles.

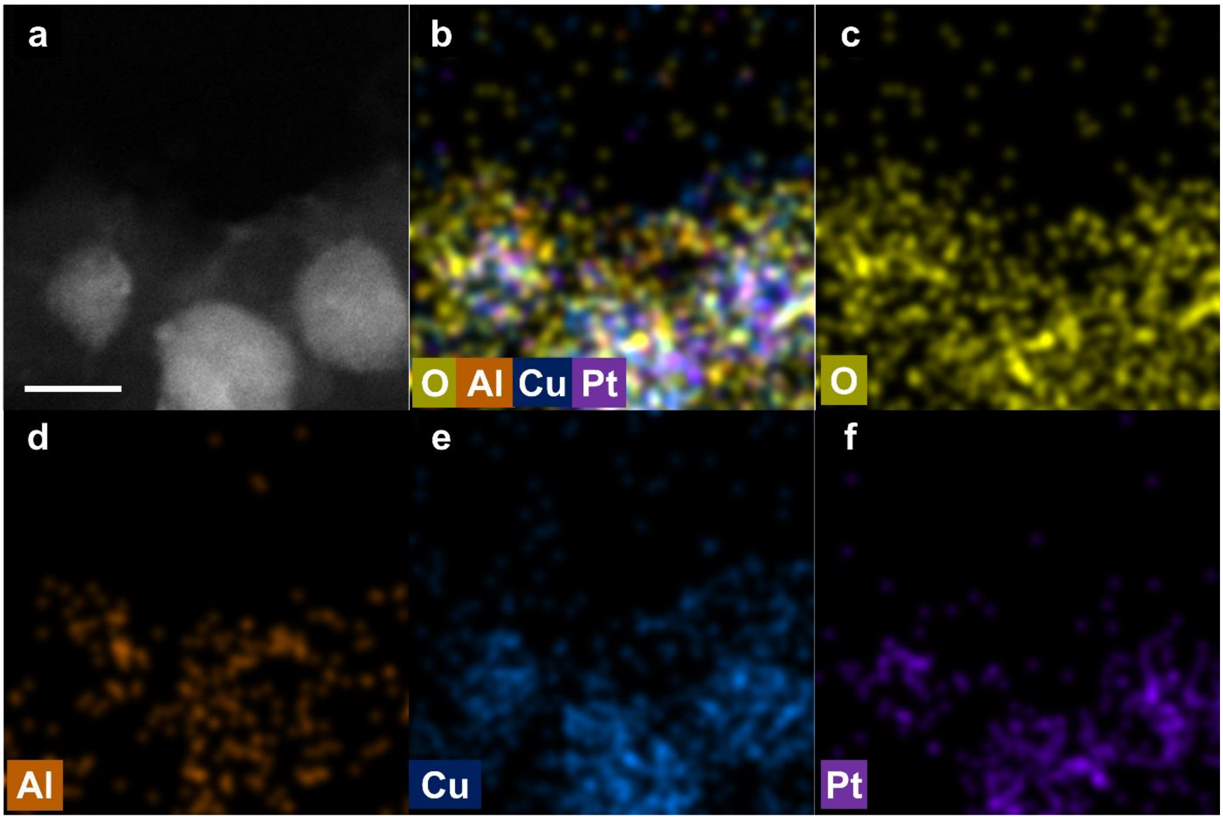

Supplementary Figure 29 | Elemental mapping of the 0.1Pt10Cu/Al_2O_3 catalyst after five successive dehydrogenation-regeneration cycles at 600 ℃. (a) HAADF STEM image of the 0.1Pt10Cu/Al_2O_3 catalyst. (b) EDS of the sample. (c) O, (d) Al, (e) Cu and (f) Pt elemental maps obtained from the same region on the sample. Scale bar, 10 nm (a).

As shown in Supplementary Figure 29, EDS of the 0.1Pt10Cu/Al_2O_3 catalyst indicates that Pt was mainly distributed on the Cu nanoparticles and not on the Al_2O_3 support by comparing the elemental maps of Pt and Cu.

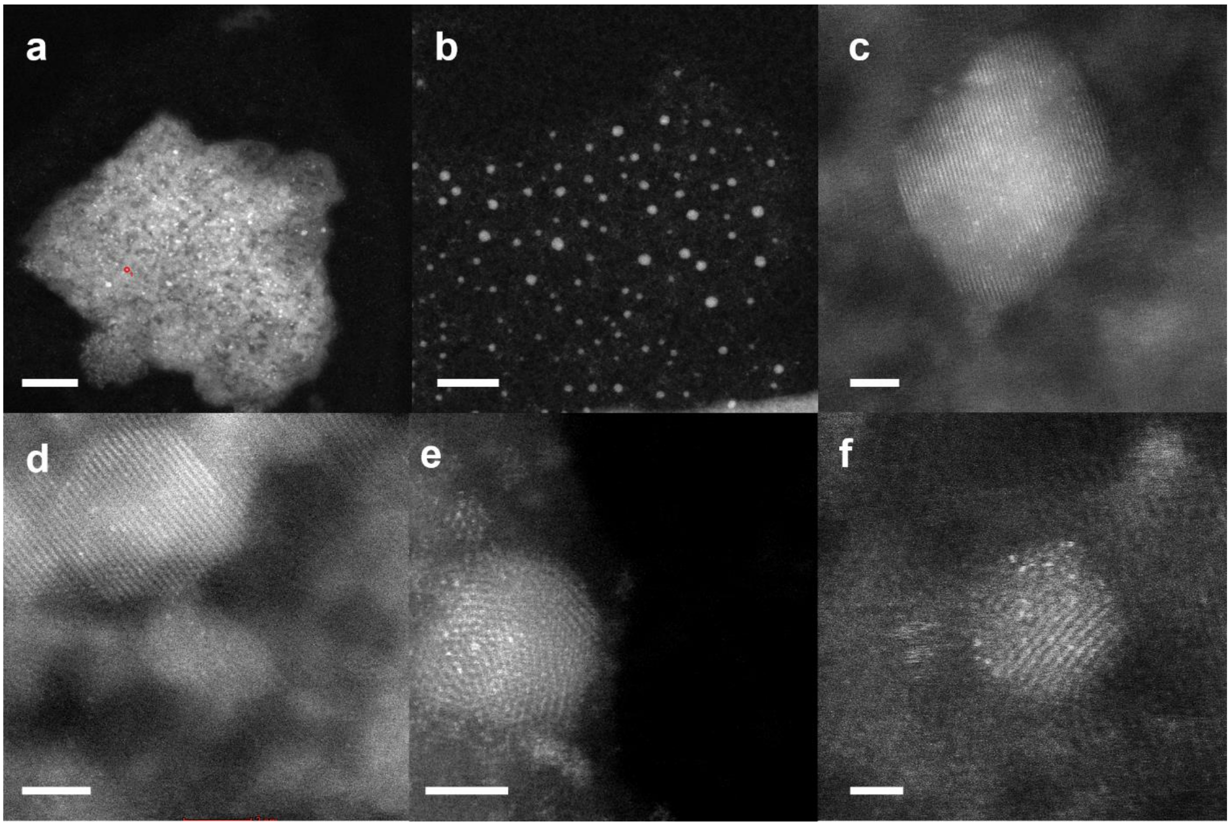

Supplementary Figure 30 | Morphology of the 0.1Pt10Cu/Al_2O_3 catalyst after five successive dehydrogenation-regeneration cycles at 600 ℃. (a-f) HAADF-STEM images with typical region of the 0.1Pt10Cu/Al_2O_3 catalyst. (c-f) The enlarged images showing Pt atoms individually dispersed on Cu nanoparticles clearly. Scale bars, 50 nm (a), 20 nm (b), 2 nm (c), (d), (e) and (f).

Since Pt atoms are brighter than Cu atoms in the dark field STEM images, single Pt atoms identified from their higher brightness comparing to their surrounding area are dispersed on the Cu nanoparticles (Supplementary Figure 30).

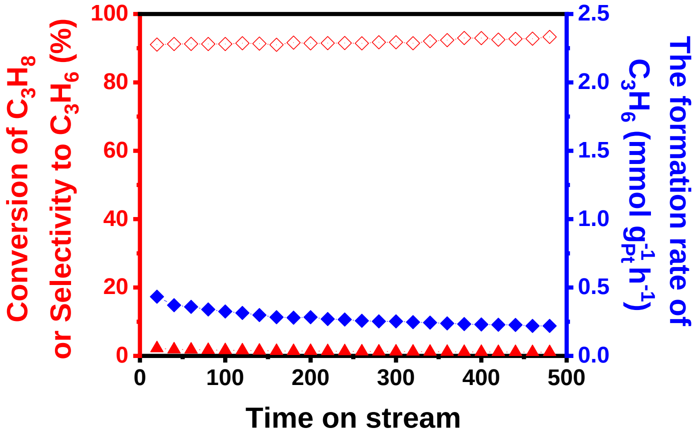

Supplementary Figure 31 | Catalytic performances of the 10Cu/Al_2O_3 catalyst.. Catalytic test conditions: atmospheric pressure, 520 ℃, WHSV of propane = 4 h^{-1}, 250 mg of sample, C_3H_8/H_2 = 1/1, with balance N_2 for total flow rate of 50 mL min^{-1}.

The 10Cu/Al_2O_3 catalyst exhibited very low activity, which can be neglected compared to the 0.1Pt10Cu/Al_2O_3 system.

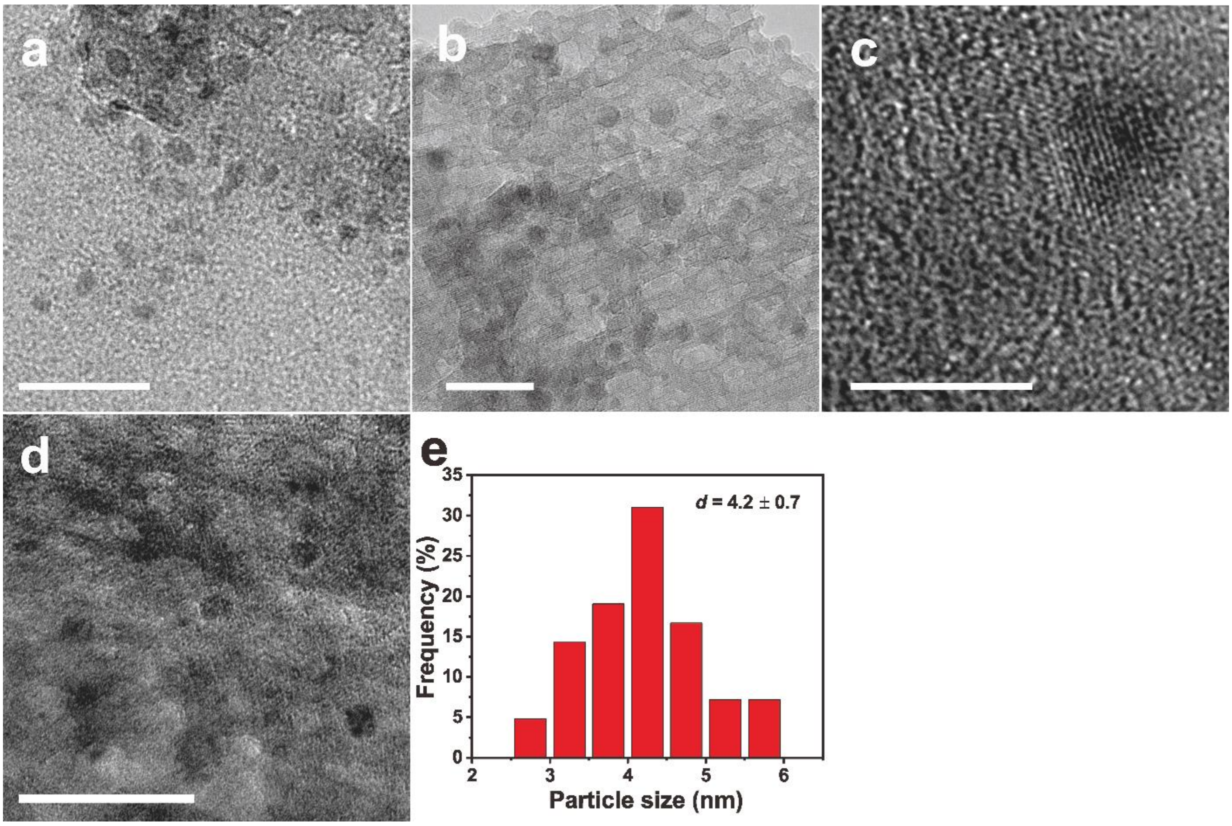

Supplementary Figure 32 | Morphology of the 0.1Pt10Cu/Al_2O_3 catalyst after reaction at 520 ℃ for 120 h. Scale bars, 20 nm (a) and (d), 10 nm (b), 5 nm (c). Catalytic test conditions: atmospheric pressure, 520 ℃, WHSV of propane = 4 h^{-1}, 250 mg of sample, C_3H_8/H_2 = 1/1, with balance N_2 for total flow rate of 50 mL min^{-1}.

The slightly increased particle size of the bimetallic nanoparticles on Al_2O_3 after reaction indicates the thermal stability of the catalyst at 520 ℃ (Supplementary Figure 32).

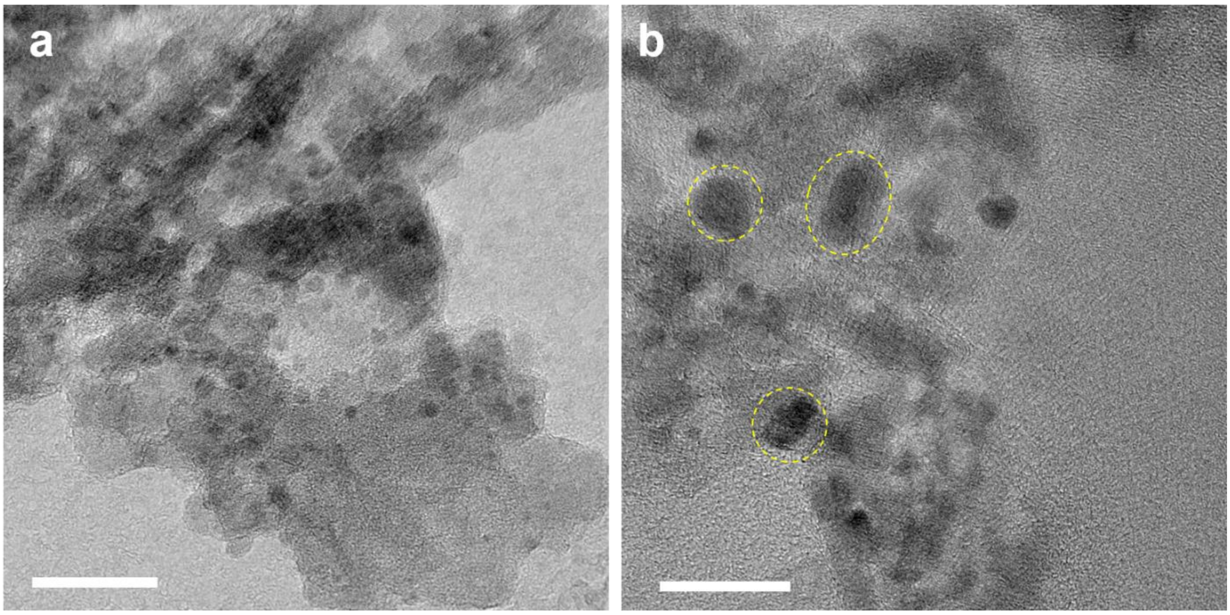

Supplementary Figure 33 | Morphology of the 0.1Pt10Cu/Al_2O_3 catalyst before and after reaction. (a) TEM image of 0.1Pt10Cu/Al_2O_3 after pre-reduction at 600 ℃ for 1 h. (b) TEM image of 0.1Pt10Cu/Al_2O_3 after reaction at 600 ℃ for 4 h. Scale bars, 20 nm (a) and (b).

When we raised the reaction temperature to 600 °C, the sintering of copper nanoparticles was obvious, which may contribute to the deactivation of the 0.1Pt10Cu/Al_2O_3 catalyst at 600 °C. Now, we are studying the stabilization of copper nanoparticles at high temperature by confinement effects.

Supplementary Table 1 | Calculated propylene adsorption energy (eV) over Pt(111), $Pt_3Cu(111)$ and Pt/Cu SAA

Slab	Propylene adsorption mode	
	di-σ	π
Pt(111)	-1.10	-0.84
$Pt_3Cu(111)$	-0.88	-0.62
Pt/Cu SAA	N.A.[a]	-0.57

[a]: N.A.: not applicable.

Supplementary Table 2 | Relative stability (eV) of single atom Pt with Cu surfaces and surface energies of clean Cu surfaces.

Slab	Surface Energy[a] eV $Å^{-2}$	Pt location	
		Surface	Subsurface
Cu(111)	0.082	0	+0.18 (+0.04[b])
Cu(100)	0.092	+0.25	+0.14
Cu(211)	0.102	+0.40	+0.22

[a]: Ref: R. Tran, Z. Xu, B. Radhakrishnan, D. Winston, W. Sun, K. A. Persson, S. P. Ong, *Surface Energies of Elemental Crystals*, Scientific Data, 2016, 3:160080, doi: 10.1038/sdata.2016.80.

[b]: Calculated with (2x2) unit cell.

Supplementary Table 3 | Relative stability (eV) of single atom Pt on Cu nanoparticles

diameter (nm)	size of nano-particle	Shape	Pt location				
			111	100	edge	corner	Subsurface of (111)
1.1	79	truncated octahedra	0.00	N.A.[a]	-0.08	0.23	-0.09
1.5	147	cuboctahedra	0.00	0.13	0.16	0.26	0.05
1.6	201	truncated octahedra	0.00	0.28	0.25	0.30	0.15
2.0	309	cuboctahedra	0.00	-0.09	0.01	0.31	-0.07
2.1	405	truncated octahedra	0.00	0.30	0.27	0.35	0.12
2.6	711	truncated octahedra	0.00	0.16	0.22	0.31	0.12

[a]: N.A.: not applicable.

Supplementary Table 4 | Relative stability (eV) of dimer Pt on Cu nanoparticles (> 2.1 nm)

diameter (nm)	size of nano-particle	Two Pt atoms on Cu particles	
		Separated Pt atoms on Cu（111）	Surface dimers on Cu（111）
2.1	405	0.00	0.14
2.6	711	0.00	0.10

Supplementary Table 5 | Calculated top adsorbed CO frequency (cm^{-1}) over Pt(111), $Pt_3Cu(111)$ and Pt/Cu SAA and experimentally measured frequencies.

Calculations		Experiments	
Slab	$\nu_{C\text{-}O}$ / cm^{-1}	Sample	$\nu_{C\text{-}O}$ / cm^{-1}
Pt(111)	2061	0.1Pt	2068
Pt/Cu SAA	2021	0.1Pt6.7Cu	2018

Supplementary Table 6 | Relative stability (eV) of single atom Pt with Ag surfaces

Slab	Pt location	
	Surface	Subsurface
Ag(111)	0	-0.15
Ag(100)	0	-0.39
Ag(211)	0	-0.52

Supplementary Table 7 | XANES edge energies and EXAFS fitting results at the Pt L3 edge

Sample	XANES edge energy (eV)	Scattering Pair	Coordination number	R^a (Å)	$\Delta\sigma^2$ ^{b}(Å^2)	E_0^c (eV)
0.1Pt6.7Cu/Al_2O_3	PtL3: 11564.6	Pt-Pt	0	--	--	--
		Pt-Cu	4.4	2.53	0.004	-7.7
		Pt-O	1.4	2.04	0.004	1.9
0.1Pt/Al_2O_3	PtL3: 11564.4	Pt-Pt	4.4	2.73	0.001	-3.6
		Pt-O	1.6	2.03	0.001	0.4
Pt Foil	PtL3: 11564.0	Pt-Pt	12	2.77	0.000	0
0.1Pt-6.7Cu-Al_2O_3	Cu K: 8979.0	Cu-Cu	7	2.55	0.001	-0.2
Cu Foil	Cu K: 8979.0	Cu-Cu	12	2.56	0.000	0.0

a. R, distance between absorber and backscattered atoms.

b. Change in the Debye–Waller factor value relative to the Debye–Waller factor of the reference compound;

c. Inner potential correction to account for the difference in the inner potential between the sample and the reference compound.

Supplementary Table 8 | Physicochemical parameters of 0.1Pt/Al_2O_3, 0.1Pt10Cu/Al_2O_3, and 10Cu/Al_2O_3.

Catalysts	Loading[a] (wt %)		S_{BET}[b] (m^2 g^{-1})	V_t[b] (cm^3 g^{-1})	Average pore diameter[b] (nm)	Dispersion of Pt (%)	Particle size of Pt (nm)
	Pt	Cu					
0.1Pt/Al_2O_3	0.10	--	144	0.22	4.2	29[c]	2.3 ±0.4[d]
0.1Pt10Cu/Al_2O_3	0.10	9.7	130	0.19	4.2	100[e]	atom size[e]
10Cu/Al_2O_3	--	9.5	129	0.19	4.2	--	--

a. Determined by ICP-OES.

b. Calculated from N_2 adsorption-desorption isotherms.

c. Measured by H_2-O_2 titration experiments. The particle size and dispersion can match very well based on a hemisphere model considering that the Pt particles are partly embedded in the support (shown in Supplementary Figure 8c).

d. Determined by the TEM images.

e. Combining the relative stability of single Pt atom on Cu nanoparticles based on DFT calculations and CO-DRIFTS, AC-HAADF-STEM images and EXAFS results, we presume that isolated Pt atoms are mainly dispersed on the surface of Cu nanoparticles.

Supplementary Table 9 | Catalytic properties of propane dehydrogenation over 0.1Pt/Al_2O_3 and 0.1Pt10Cu/Al_2O_3.

Catalysts	T (°C)	Conversion of C_3H_8 (%)	Selectivity to C_3H_6 (%)	Specific activity of C_3H_6 formation[(d)] (s^{-1})	k_d[(e)] (h^{-1})	τ[(f)] (h)
0.1Pt/Al_2O_3	520	5.8(2.6) [(b)]	70(68) [(b)]	0.21	0.070	14
	550	13.6(5.0) [(b)]	68(69) [(b)]	0.45	0.091	11
0.1Pt10Cu/Al_2O_3	520	13.1(12.4) [(c)]	87(89) [(c)]	0.56	5E-4	2E4
	550	24.1(21.6)[(b)]	88(90) [(b)]	0.95	0.012	83

a. Atmospheric pressure, WHSV propane = 4 h^{-1}, 250 mg of catalyst, C_3H_8/H_2 = 1/1, with balance N_2 for total flow rate of 50 mL min^{-1}.

b. The values outside and inside the brackets are the data obtained at 20 min and 12 h.

c. The values outside and inside the brackets are the data obtained at 20 min and 120 h.

d. Moles of C_3H_6 per mole Pt atom per second at 20 min.

e. k_d, deactivation rate constant, calculated from $\ln [(1-X_{final})/X_{final}] = k_d t + \ln [(1-X_{initial})/X_{initial}]$.

f. Time required for rates to decrease by e^{-1}, $\tau = 1/k_d$.

Supplementary Table 10 | Catalytic properties of propane dehydrogenation over some representative Pt-based catalysts.

No	Catalysts	T (℃)	WHSV (h^{-1})	Feed composition	Conv (%)[(b)]	Sel (%)	Specific activity of C_3H_6 formation (s^{-1})[(c)]	k_d[(d)] (h^{-1})	τ[(e)] (h)	Ref
1	0.9wt%Pt/ Mg(Ga)(Al)O	600	2.6	C_3H_8=20,H_2 =25,He=55	16-11.4	99.2	0.0113	0.20	5	4
2	0.35wt%Pt-1.26wt% Sn/Al_2O_3	519	3.5	C_3H_8=30, N_2=70	31-20	95-98	0.109	0.29	3.4	5
3	0.5wt%Pt-Sn-Na/Al-SBA-15	590	3.0	C_3H_8=75, H_2=25.	27.5- 12.6	~94	0.143	0.024	41.7	6
4	0.5wt%Pt/Mg(Sn) (Al)O@Al_2O_3	600	14	C_3H_8/H_2/Ar =1/0.5/2	48.3-43.0	86.4-98.1	1.44	0.0045	224	7
5	0.3wt%Pt-0.2wt%Sn -0.5wt%K/Al_2O_3	600	4	C_3H_8/H_2= 1/0.5	39.9-38.2	92.0-95.5	0.602	0.0029	349	8
6	0.46wt%Pt-0.83wt% Sn/15MAF	590	3	C_3H_8/H_2= 1/0.25	24.7-23.5	92.5-99.5	0.184	0.0094	106	9
7	0.5wt%Pt-0.6wt%Sn /TS-1	590	3	C_3H_8/H_2/ N_2=1/1/4	53.5-47.7	92.5-93.0	0.40	0.033	30.3	10
8	1wt%Pt-2wt%Sn/2 Mg-SBA-15	580	8.25	C_3H_8/Ar= 7/3	43.0-38.1	97.8	0.427	0.034	29.4	11
9	0.5wt%Pt-2.0wt%Sn -1.0wt%Na/SUZ-4	590	3	C_3H_8/H_2= 1/3	24-22	90-91	0.160	0.011	88	12
10	0.5wt%Pt-Zn/Na-Y	555	2.6	C_3H_8=100	24.8-15.7	91.6-90.6	0.145	0.048	20.8	13
11	0.6wt%Pt-5wt%Ga/	605	3.9	C_3H_8=73,	31-30	97-98	0.176	0.024	41.7	13

	$MgAl_2O_4$			H_2=27						
12	0.7wt%Pt/ Mg(In)(Al)O	600	2.6	C_3H_8=20,H_2 =25,He=55	20.4-16.3	98	0.183	0.14	7.1	[14]
13	0.5wt%Pt-Na/ Sn-ZSM-5	590	3	C_3H_8=75, H_2=25	41.7-39.1	95.3-98	0.220	0.012	83.3	[15]
14	0.5wt%Pt-0.6wt% Sn/$MgAl_2O_4$	550	36.6	C_3H_8=50, H_2=50	12-11	92-95	0.497	0.033	30.3	[16]
15	0.1wt%Pt-Na-[Fe]/ZSM-5	520	15.1	C_3H_8=25, He=75	33-13	98.3-99.8	1.51	0.008	125	[17]
16	0.36wt%Pt-0.68wt%Sn/ Al_2O_3-sheet	590	9.4	C_3H_8=16, H_2=20, N_2=64	48.7-44.6	98.7	1.58	0.007	143	[18]

a. The catalsts included here are only the best performing ones from the articles considered.

b. First and second values are obtained at the start and end of the cycle.

c. Specific activity is defined as the moles of C_3H_6 formation per mole Pt atoms per second.

d. k_d, deactivation rate constant is calculated from $\ln[(1-X_{final})/X_{final}] = k_d\, t + \ln[(1-X_{initial})/X_{initial}]$.

e. Time required for rates to decrease by e^{-1}, $\tau = 1/k_d$.

Supplementary References

1. Jiang, F., Zeng, L., Li, S., Liu, G., Wang, S., Gong, J. Propane dehydrogenation over Pt/TiO_2–Al_2O_3 catalysts. *ACS Catal.* 2015, **5**(1)**:** 438-447.

2. Ma, Z., Wu, Z., Miller, J.T. Effect of Cu content on the bimetallic Pt–Cu catalysts for propane dehydrogenation. *Catal. Struct. React.* 2017, **3**(1-2)**:** 43-53.

3. Luo, M.-F., Fang, P., He, M., Xie, Y.-L. In situ XRD, Raman, and TPR studies of CuO/Al_2O_3 catalysts for CO oxidation. *J. Mol. Catal. A. Chem.* 2005, **239**(1-2)**:** 243-248.

4. Siddiqi, G., Sun, P., Galvita, V., Bell, A.T. Catalyst performance of novel Pt/Mg(Ga)(Al)O catalysts for alkane dehydrogenation. *J. Catal.* 2010, **274**(2)**:** 200-206.

5. Bariås, O.A., Holmen, A., Blekkan, E.A. Propane dehydrogenation over supported Pt and Pt–Sn catalysts: Catalyst preparation, characterization, and activity measurements. *J. Catal.* 1996, **158**(1)**:** 1-12.

6. Duan, Y., Zhou, Y., Zhang, Y., Sheng, X., Xue, M. Effect of sodium addition to PtSn/Al-SBA-15 on the catalytic properties in propane dehydrogenation. *Catal. Lett.* 2011, **141**(1)**:** 120-127.

7. Zhu, Y., An, Z., Song, H., Xiang, X., Yan, W., He, J. Lattice-confined Sn (IV/II) stabilizing raft-like Pt clusters: High selectivity and durability in propane dehydrogenation. *ACS Catal.* 2017, **7**(10)**:** 6973-6978.

8. Shi, Y., Li, X., Rong, X., Gu, B., Wei, H., Sun, C. Influence of support on the catalytic properties of Pt–Sn–K/θ-Al_2O_3 for propane dehydrogenation. *RSC Adv.* 2017, **7**(32)**:** 19841-19848.

9. Zhang, H., Zhang, Y., Zhou, Y., Sheng, X., Zhang, C., Fang, J., *et al.* Morphology-controlled fabrication of biomorphic alumina-based hierarchical LDH compounds for propane dehydrogenation reaction. *New. J. Chem.* 2018, **42**(1)**:** 103-110.

10. Li, J., Li, J., Zhao, Z., Fan, X., Liu, J., Wei, Y., *et al.* Size effect of TS-1 supports on the catalytic performance of PtSn/TS-1 catalysts for propane dehydrogenation. *J. Catal.* 2017, **352:** 361-370.

11. Li, B., Xu, Z., Jing, F., Luo, S., Chu, W. Facile one-pot synthesized ordered mesoporous Mg-SBA-15 supported PtSn catalysts for propane dehydrogenation. *Appl. Catal. A. Gen.* 2017, **533:** 17-27.

12. Zhou, H., Gong, J., Xu, B., Yu, L., Fan, Y. PtSnNa@SUZ-4-catalyzed propane dehydrogenation. *Appl. Catal. A. Gen.* 2016, **527:** 30-35.

13. De Cola, P.L., Gläser, R., Weitkamp, J. Non-oxidative propane dehydrogenation over Pt–Zn-containing zeolites. *Appl. Catal. A. Gen.* 2006, **306:** 85-97.

14. Sun, P., Siddiqi, G., Vining, W.C., Chi, M., Bell, A.T. Novel Pt/Mg(In)(Al)O catalysts for ethane and propane dehydrogenation. *J. Catal.* 2011, **282**(1)**:** 165-174.

15. Zhang, Y., Zhou, Y., Huang, L., Xue, M., Zhang, S. Sn-modified ZSM-5 as support for platinum catalyst in propane dehydrogenation. *Ind. Eng. Chem. Res.* 2011, **50**(13)**:** 7896-7902.

16. Salmones, J., Wang, J.-A., Galicia, J.A., Aguilar-Rios, G. H_2 reduction behaviors and catalytic performance of bimetallic tin-modified platinum catalysts for propane dehydrogenation. *J. Mol. Catal. A. Chem.* 2002, **184**(1–2)**:** 203-213.

17. Waku, T., Biscardi, J.A., Iglesia, E. Active, selective, and stable Pt/Na-[Fe]ZSM-5 catalyst for the dehydrogenation of light alkanes. *Chem. Commun.* 2003(14)**:** 1764-1765.

18. Shi, L., Deng, G.M., Li, W.C., Miao, S., Wang, Q.N., Zhang, W.P., *et al.* Al_2O_3 nanosheets rich in pentacoordinate Al(3+) ions stabilize Pt-Sn clusters for propane dehydrogenation. *Angew. Chem. Int. Ed.* 2015, **54**(47)**:** 13994-13998.
